# Inferring individual sexual action dispositions from egocentric network data on dyadic sexual outcomes

**DOI:** 10.1371/journal.pone.0207116

**Published:** 2018-11-12

**Authors:** Disa Hansson, Veronika Fridlund, Karin Stenqvist, Tom Britton, Fredrik Liljeros

**Affiliations:** 1 Department of Mathematics, Stockholm University, Stockholm, Sweden; 2 Department of Sociology, Stockholm University, Stockholm, Sweden; 3 Section of Social Medicine, University of Gothenburg, Gothenburg, Sweden; University of Westminster, UNITED KINGDOM

## Abstract

In this paper we present a family of models that allows us to estimate egos’ unobserved action dispositions from a joint behavioural outcome of a dyadic social interaction process of both egos’ and alters’ action dispositions. The method is put to test on a data set containing two different types of dyadic activities of high relevance for the spread of sexually transmitted infections (STI), condom use and anal sex. The data consists of individuals older than 15 years old who visited one of the nine youth clinics in the Vastra Gotaland region of Sweden between February 2010 and March 2011 for STI testing. This is hence a group of special interest for STI interventions. We cannot find any difference in condom disposition between women and men. Condoms are initially used more often in less risky types of relationships, especially if the partner ends up as a main partner. When studying the disposition towards anal sex we do however find a difference between men and women. Women are more against practising anal sex than men while the majority of men are neutral towards anal sex.

## Introduction

Sexual infections are a major public health problem in modern societies. Information campaigns, with the purpose of reducing sexual risk-taking such as unprotected sex, have not achieved the desired effect [1]. Unsuccessful information campaigns are not unique to sexual risk-taking [2]. Experiences from similar campaigns targeting other types of health related risk behaviours, such as smoking and dietary habits, shows that information efforts with the aim to change the lifestyle of individuals commonly have very little or no effect at all.

Recently, two linked explanations concerning why the above described campaigns proved to have such a poor effect have been advanced [3]. The first explanation is that these types of campaigns are often based on the oversimplified notion that human behaviour is exclusively governed by a subject who knows what it wants to achieve and consciously strives to achieve it. Modern cognitive science shows that behaviours that often are interpreted as intentional actions largely consist of socially learned unreflected behaviour: a type of unconscious automatic habitual behaviour. Behaviour caused by socially inherited behavioural dispositions executed through fast associative thinking, has recently in popularised form been described to a wider community by David Kanneman as fast thinking.

The awareness of the existence of this type of behaviour is not new in sociology. It constitutes what has been a central element in European sociology since the 1970s and can be found in Gidden’s [4] theory of society in his concept of routinised behaviour as well as in Bourdieu’s concept of habitus [5]. In research on sexual behaviour similar ideas are found in Gagnon and Simon’s theories of sexual scripts [6].

Habits we take for granted would require other types of tools to change than reflexive intentional behaviour. What these tools are is not obvious, a clue to the solution may be found in the second explanation for the observed poor effects of intervention campaigns. The second explanation is that the simplistic image of human actions misses the fact that the causes of human habits often originate from the individual’s observations of how others behave, rather than from the individual’s own conscious decisions. When considering the importance of social influences for habitual behaviour and risk behaviour in general, it becomes obvious that we need a deeper knowledge based on the individual’s actual behaviour and the role social influence has in forming this specific type of behaviour.

Sexual risk-taking differs in some key points from other health-related risk behaviours. The most important difference is that the very act in itself consists of an interaction between two individuals. The fact that at least two individuals must be involved means that there will always be an element of social influence between the involved actors. If the population under consideration is heterosexual, the behaviour takes place between two individuals of the opposite sex. Since we are all constantly exposed to different ideals for male and female sexuality by friends, the media and pornography, the experience of using condoms and practising anal sex is likely to differ between men and women.

When sexual behaviour data is gathered, it is usually only from one of the partners, giving rise to egocentric network data: information concerning sampled individuals, the egos; and via these limited information of the individuals connected to the egos, named alters [7].

In this paper we derive new statistical methods for egocentric network data that allows us to estimate the unobservable action dispositions where the interactions constitute the direct outcome of two individuals’ dispositions. The method is put to test on a data set of 673 participants and their sexual behaviour [8]. Two different types of dyadic activities of high relevance for the spread of sexually transmitted infections (STI) are analysed, condom use and anal sex.

There are contradicting results reported concerning gender differences regarding the attitude towards condom use. Several studies report a male resistance to condom use [8–11], whereas a Swedish study reported that more men than women did not use a condom because their partner was against it [12]. Therefore we examine if the condom dispositions of men and women differ. We also study if dispositions differ between different relationship types.

## Materials and methods

### Description of the data

Sexual behaviour data was gathered of sexually active youths who visited one of nine out of 55 youth clinics in the Västra Götaland region of Sweden between February 2010 and March 2011 for STI testing. The sample is therefore not representative of the whole population; however, this is a very interesting group from an epidemiological viewpoint.

From the beginning there were 673 individuals in the data set. There are very few homosexuals in the data; therefore, we chose to only look at the heterosexual subset. For each individual the information used in the analysis concerns the following: number of sex partners the last year and for each partner their relationship type, if a condom was used in the first sexual contact and if they had anal sex. Sexual contacts of such type that a condom could not be used (non-penetrative sex) were removed when studying condom dispositions. Four different kinds of relationship types could be chosen (Table 1), note that the relationship type could have been determined after the first sexual contact.

**Table 1:** Summary of information used in analysis. The two subsets used are taken from the sample of 673 participants.

	Condom use data			Anal sex data		
Participants	Total	Male	Female	Total	Male	Female
	645	224 (35%)	421 (65%)	646	224 (35%)	422 (65%)
Age						
Min	15	15	15	15	15	15
Max	26	26	25	26	26	25
Mean	20	20	20	20	20	20
Standard deviation	2.39	2.43	2.35	2.34	2.43	2.29
Number of partners						
Mean	3.11	3.05	3.14	3.14	3.07	3.18
Standard deviation	1.94	1.99	1.91	1.96	1.97	1.95
Max	13	13	11	13	13	11
Participant practiced anal sex	-	-	-	277 (43%)	102 (46%)	157 (37%)
Partners among participants	Total	Of male	Of female	Total	Of male	Of female
	2008	684	1324	2029	689	1340
Condom first sexual encounter	632 (31%)	206 (30%)	426 (32%)	-	-	-
Anal sex with partner	-	-	-	441 (22%)	166 (24%)	275 (21%)
Type of relationship						
Main	538 (27%)	166 (24%)	372 (28%)	541 (27%)	166 (24%)	375 (28%)
Regular partner	376 (19%)	118 (17%)	258 (19%)	382 (19%)	119 (17%)	263 (20%)
Casual known partner	640 (32%)	218 (32%)	422 (32%)	646 (32%)	221 (32%)	425 (32%)
Casual unknown partner	454 (23%)	182 (27%)	272 (21%)	460 (23%)	183 (27%)	277 (21%)

Due to the sensitive nature of the questions asked, participants did not need to answer every question if they did not feel comfortable. Therefore, there are missing values for some of the participants. To analyse condom disposition, we used a subset of 645 participants who gave information of their condom use. To analyse anal sex disposition we used a subset of 646 participants who gave information of their anal sex behaviour. A summary of the two subsets of data used, condom use and anal sex data, are given in Table 1.

### Study approval

This study was approved by The Regional ethical review board in Gothenburg (file number 637-09).

At the youth clinics, individuals belonging to the study’s target group were given oral and written information concerning the study from the staff. The information given concerned the background and purpose of the study, what it meant to participate, and who was responsible for the study. It was also clear from the information that participating was voluntary and that you could cancel the study at any time. The participant was also informed that the youth clinic staff would not be able to view the questionnaire and that if the person chose to abstain or cancel the study, this would not affect the person’s continued contact with the youth clinic.

Written consent was obtained from the participants. The ethics committee approved that parental consent was not required for youths of the age 15 and older. The youths that gave their consent to participate was given a questionnaire at the youth clinic. The participants did not specify their identity on the questionnaires, and to ensure the anonymity of a participant the questionnaire was answered in a secluded space. The participant then put the questionnaire in an envelope which in turn was put in a locked locker. On another paper the participant wrote his or her email address which was put in another envelope in the same locker. The email address was saved in order to send out a follow-up questionnaire without being able to connect the participant to the first questionnaire. Instead we had six background questions which the participant wrote on all questionnaires to be able to connect the questionnaires without knowing the identity of the participant.

### Model of disposition to sexual outcome

In the descriptions of the models we will consider condom use only, but the exact same method is used to analyse the anal sex dispositions. Two different types of outcomes exist, a condom-contact and a non-condom contact. Each individual has a condom disposition and the outcome between two individuals having sex is determined by the two individuals’ condom disposition. Our assumptions concerning the dispositions and independence will now be outlined. Individuals’ dispositions are independent of each other and an individual uses the same disposition with all its partners. The condom disposition of an individual does not affect the number of partners of that individual. Additionally, an individual’s choice of partner is not affected by which disposition the individual or the potential partner has. In the first sexual contact between two individuals this is a valid assumption, they would normally not have had the time to influence each other much. Since the data sample process did not include contact tracing individuals will be treated as independent of each other. If contact tracing was included an individual would have been both a sampled individual and a partner of another sampled individual.

The dispositions are not observable in the data; only the sexual outcome is observed. The aim is to model the interaction of two individuals’ disposition and how they affect whether or not a condom is used in order to determine the individual condom dispositions. The models derived may determine if there is a difference in disposition between genders in two different aspects. First, if there is a difference in disposition distribution between males and females, a difference which may have risen from different ideals. The second aspect the model is able to capture is if the disposition of one of the sexes weighs higher with regard to which outcome we observe. Also, we derive models to test if individuals use condom more often when having casual sexual relationships compared to steady relationships.

We will now describe the models for condom use in the first sexual contact between two individuals. Henceforth we will study the non-condom dispositions, since we chose to code a person who never wants to use a condom as ‘1’ and a person who always want to use a condom as ‘0’. Each individual *i* has a non-condom disposition *x*_*i*_, 0 ≤ *x*_*i*_ ≤ 1, for *i* = 1, …, *n* where *n* is the sample size. To model condom use we need a translation of a couple’s two dispositions into a probability of condom use.

#### Geometric mean model

The first model states that the non-condom probability of two individuals is given by the geometric mean of the individuals’ dispositions, i.e. a condom is not used with a probability $\sqrt{x_{i}x_{j}}$. The goal was to capture the following: two individuals with the same disposition *x* should result in a condom probability *x*, and if a condom person (*x*_*i*_ = 0) meets a non-condom person (*x*_*j*_ = 1) a condom should be used. With the geometric mean these requirements are met. Within this first model, we use a beta distribution for the dispositions when analysing the data.

#### Pro-con-neutral model

The second model for the translation of non-condom dispositions to a non-condom probability captures the hypothesis that people with strong opinions, either for or against using a condom, are more influential than individuals with weaker opinions. This is accomplished by creating three dispositions, either an individual is completely for using a condom (disposition 0), against condom (disposition 1), or an individual is neutral (easily persuaded). An individual neutral towards condom who meets: a pro condom person, will use a condom; a person who does not want to use a condom, will not use a condom; another neutral individual, a condom is used with probability 0.5. If two individuals being pro condom meet, they will use a condom. If two individuals being against condom meet, they will not use a condom. If two individuals of opposite disposition meet, a condom individual and an individual against condom use, then they will use a condom. The assumption that a condom will be used if a condom person meets a non-condom person may be relaxed, resulting in an extension of the pro-con-neutral model. We then add an additional parameter *ε*_*CN*_ defined as the probability of no condom when a condom person (*C*) meets a non-condom person (*N*).

#### Pro-con model

The simplest model that will be used is a special case of both the above models. This is a model where each individual is one of two types; either pro-condom (disposition 0) or against using a condom (disposition 1), and that a condom is always used between a condom individual and a non-condom individual.

#### Gender asymmetries

The models can be extended to take gender into account. The male and female dispositions may differ in distribution. It is also possible to model whether it is the opinions of females or males that weigh higher in the condom decision. In the case of the pro-con-neutral model, this second extension is achieved by examining the different scenarios that can occur when a man and woman of opposite dispositions meet.

Let $\varepsilon_{CN}^{MW}$ denote the non-condom probability between a condom male and a non-condom female, and $\varepsilon_{NC}^{MW}$ the non-condom probability between a non-condom male and a condom female. If for example $\varepsilon_{CN}^{MW}=1$ and $\varepsilon_{NC}^{MW}=0$, women would be the ones who’s disposition decides what happens.

#### Different types of sexual relationships

The models are also extended to take two different relationship categories into account. Thus to answer questions of the type, if we merge the two casual relationships into one category and merge the regular and main relationship into one other category, is condom used more often in the casual relationship category? For derivation of the likelihoods see S1 Appendix.

## Results

The method developed is applied to the data set from [8] to study the dispositions of condom use and anal sex. The analyses are done with the R software [13]. Since the pro-con-neutral model and the geometric mean model are not generalisations of each other, AIC scores are used for model comparison [14]. The model with the lowest AIC score fit the data best.

Due to the models character, confidence intervals are not easily obtained in standard fashions. Bootstrap confidence intervals has also its problems: each observation in our data, each participant, contains a different amount of information with respect to the parameters. In trying to determine what disposition a participant has, the participant with 10 sex partners would give us a much better guess than a participant with only one sex partner.

To verify that the models give reliable estimates, we instead did a simulation study, see S2 Appendix.

### First sexual contact and condom dispositions

The best fitted models for non-condom dispositions when the genders have the same distribution and allowing them to be different are given in Table 2. For both cases the pro-con-neutral (where a condom is used in condom-noncondom pairs) fit the data best. Roughly 25% of all individuals are non-condom individuals, very few are condom individuals (4%) and the rest are neutral according to this model. Concerning relationship type we find that in the first sexual contact individuals tend to use condoms more often in to-be main relationships (p-value of order 10^−9^). Again it is the pro-con-neutral model that fit the best. The probability of condom use in the first sexual contact of a randomly chosen couple who entered main relationship is 23% higher compared to when we do not take relationship type into account (from 34% to 42%). The complete sets of results are given in S3 Appendix.

**Table 2:** Best fitted models non-condom disposition.

	Pro-con-neutral model							
	LogL	AIC	${\hat{p}}_{N}$		${\hat{p}}_{C}$		${\hat{p}}_{I}$	
Gender not taken into account	-768.0	1540.0	0.245		0.031		0.724	
			Men	Wom	Men	Wom	Men	Wom
Gender taken into account	-766.8	1541.6	0.256	0.204	0	0.041	0.744	0.755

Maximum Log Likelihood, AIC and parameter estimates for the pro-con-neutral model. We show the results for the model assuming that men and women have the same non-condom disposition distribution and the results allowing them to be different. *p*_*N*_ stands for the probability of being a non-condom person, *p*_*C*_ of being a condom person and *p*_*I*_ of being neutral.

### Anal sex dispositions

For the anal sex dispositions we find a strong indication of a difference between men and women, in Table 3 the best fitted models are shown. Assuming that men and women have the same disposition distribution the pro-con model fits the data best, and if individuals of opposite disposition meet they will have anal sex with probability 0.91. Taking gender into account, the pro-con-neutral model gives a better fit. Between this model and the best fitted model where gender difference is not taken into account (pro-con model), we do a likelihood ratio test. The model with gender differences has a *p*−value 0.0503. This *p*−value is a strong indication that there is a difference between men and women. The main difference between men and women is that more women than men are against having anal sex (77% for women in comparison to 30% for men). The estimates for all models assuming men and women draw their dispositions from the same distribution can be found in S1 Table and the estimates assuming men and women draw their dispositions from different distributions can be found in S2 Table.

**Table 3:** Best fitted models anal sex disposition.

	LogL	AIC	${\hat{p}}_{A}$		${\hat{p}}_{N}$		${\hat{p}}_{I}$		${\hat{\varepsilon }}_{NA}$	
Casual & steady	-672.5	1348.9	0.137		0.863		-		0.91	
			Men	Wom	Men	Wom	Men	Wom		
	-668.6	1347.2	0.105	0.119	0.296	0.773	0.599	0.108	0.888	
	LogL	AIC	${\hat{p}}_{A}$		${\hat{p}}_{N}$		${\hat{p}}_{I}$		${\hat{\varepsilon }}_{NA}^{MW}$	${\hat{\varepsilon }}_{AN}^{MW}$
Only Casual	-283.2	572.4	0.076		0.924		-		0.995	0.757
			Men	Wom	Men	Wom	Men	Wom		
	-279.9	571.7	0.125	0.058	0.875	0.429	0	0.513	1	0

Maximum Log Likelihood, AIC and parameter estimates for the best fitted models of anal sex dispositions. The results both assuming that men and women have the same anal sex disposition distribution and allowing them to be different are shown. *p*_*A*_ stands for the probability of being an anal sex person, *p*_*N*_ of being against anal sex and *p*_*I*_ of being neutral

Next, we conducted the analysis exclusively on the steady relationships and casual relationships, respectively, to examine if there is any difference between the two. For steady relationships we find that there is no gender difference (*p*−value 0.32, see S3 Table for estimates and likelihoods assuming men and women draw their dispositions from the same distribution and S4 Table for estimates and likelihoods assuming men and women draw their dispositions from different distributions). On the other hand, for the casual relationship data we find a difference between men and women (*p*−value 0.037). Men are either for (12.5%) or against anal sex (87.5%) (Table 3). Women can be of one of three types; for anal sex (6%), against anal sex (43%) or neutral (51%). Further, if one of the individuals having sex is for anal sex and the other against, the women will decide what happens.

Some caution should be taken when drawing conclusions from the results of the anal sex dispositions for the casual relationships. The sample size of men is quite low for the model where we let females and males have different disposition distributions. If we can reject the null model (that there is no difference) in favour of the alternative model (that there is a difference between genders) our simulation study shows that the distributions should indeed be different. However, the point estimates for are a bit more unreliable.

## Discussion

In this paper we estimate egos’ unobserved action dispositions from a joint behavioural outcome of a social interaction. This is despite the observed outcome not necessarily agreeing with the ego’s disposition, due to a partner with a different disposition. The models are able to estimate potential asymmetries between the sexes in terms of the relative strength of action dispositions; hence in some respect it is possible to say that we are able to estimate the effect of patriarchal structures in the contexts under study here.

We have used two different families of behavioural disposition models. In the pro-con model individuals either have a complete tendency for or against the sexual behaviour and the translation from the sexual dispositions to behaviour is done via the geometric mean of the two dispositions. In the pro-con-neutral model we add a category of neutral individuals who will act according to their partner’s tendency; the individual with a strong tendency in either direction will decide which outcome we observe. The models are extended to take gender differences into account as well as different types of relationships. They can also be extended in such a way that we may put more weight on any of the two genders to influence the outcome.

Two different action dispositions have been studied on a data set of heterosexual youths; non-condom dispositions and anal sex dispositions. Regarding the non-condom dispositions, the best fitted model is the pro-con-neutral model. We find that individuals behave differently in the first sexual contact depending on which type of relationship the two individuals will come to have. Condoms are initially used more often in relationships where the partner ends up as a main partner. Note that two individuals who later become main partners may not know this the first time they have sex. Possible explanations for this result could be that with a to-be main partner the first sexual contact is more planned than in a casual relationship and therefore the condom exist there physically; you are more considerate towards your partner and feel secure enough to bring up the question of contraceptives. Another explanation could be that a certain type of individual has main partners to a larger extent.

We cannot find any difference in non-condom disposition between women and men in this study of Swedish youths. Our simulation study revealed that if there was a difference in disposition distribution between women and men we would be able to detect this. This is a very important finding since most studies report a stronger will among women toward condom use. A previous study on the same data set showed a discrepancy between an actual willingness to use a condom and condom use among women [8]. This demonstrates the importance of focusing on behavioural dispositions estimated from observed or reported data instead of reported attitudes. It is possible that the most effective and perhaps only strategy to increase condom use is to focus on the time in life when the behavioural dispositions towards contraception methods are formed, i.e. at the time of the sexual debut.

For anal sex we find a strong indication that men and women have different dispositions (*p*−value 0.0503), more women than men are against having anal sex. For casual relationships the gender differences are significant (*p*−value 0.037). Men can be one of two types, either for (12%) or against (88%) anal sex, whereas women can either be for (6%), neutral (51%) or against (43%) anal sex. Additionally, if one of the individuals having sex is for anal sex and the other against, the woman will decide what happens.

## Supporting information

S1 Appendix Derivation of likelihoods. (PDF)

S2 Appendix Simulation results. (PDF)

S3 Appendix Additional results for first sexual contact and condom dispositions. (PDF)

S1 Table Anal sex disposition results assuming men and women draw their dispositions from the same distribution. (PDF)

S2 Table Anal sex disposition results assuming females and males draw their disposition from different distributions. (PDF)

S3 Table Anal sex disposition results for steady relationships assuming men and women draw their dispositions from the same distribution. (PDF)

S4 Table Anal sex disposition results for steady relationships assuming men and women draw their dispositions from different distributions. (PDF)

S5 Table Condom use data. (XLSX)

S6 Table Anal sex data. (XLSX)
