# Supplementary material for: Inferring individual sexual action dispositions from egocentric network data on dyadic sexual outcomes
Source: PLoS One. 2018 Nov 12;13(11):e0207116. doi: 10.1371/journal.pone.0207116 (PMC6231623; doi:10.1371/journal.pone.0207116)
Supplement: S1 Appendix — (PDF) [file pone.0207116.s001.pdf]

Appendix S1: Derivation of Likelihoods

We will here give a more thorough explanation of the models and the equations used to obtain the likelihood equations, which is needed in order to do inference.

Each individual in the data set represents a node, a sexual relationship between two individuals represents an edge between these nodes, and the number of sexual contacts during the last year d is the degree of that node. There exist two different types of sexual contacts, a condom-contact and a non-condom contact. For each model we need to find the probability to observe an individual with k non-condom contacts among d contacts in total. The methods is different depending on which rule we assign to get a condom probability from the two meeting individuals' dispositions.

S1.1 Dispositions according to an arbitrary distribution with the geometric mean rule

In this section we will derive the model for the first sexual contact with an arbitrary distribution with support on $[0, 1]$ for the non-condom dispositions. Since it is assumed that individuals are independent of each other, the likelihood will be the product of the probability of observing each node.

Let each individual i have a non-condom disposition x_i, $0 \leq x_i \leq 1$, where $i = 1, ..., n$ and n is the sample size. Each individual will use the same non-condom disposition in all contacts. If an individual has the disposition $x_i = 0$ he or she wants to use a condom by all means, and if the disposition is $x_i = 1$ he or she does not want to use a condom. We want the rule for the non-condom probability of the two individuals' non-condom dispositions to be rather simple, and our goal is to capture the following: if two individuals with the same disposition x meet, the combined probability to use a condom should be x. Additional to this, if a condom person meets a non-condom person, a condom should be used. With the geometric mean for the non-condom probability these requirements are met.

When two individuals meet their combined dispositions will via the geometric mean decide whether they use a condom or not. A condom is not used with a probability $\sqrt{x_i x_j}$ and a condom is used with a probability $1 - \sqrt{x_i x_j}$. Each non-condom disposition, x_i $i = 1, 2, ..., n$, is drawn from an underlying random variable X, which distribution we aim to infer. The observed data is whether or not a condom was used and the individual dispositions are not observed. Let Y_{ij} denote the indicator function of whether or not a condom was not used in a sexual meeting between i and j, given the dispositions of two individuals we then have that

$$Y_{ij}|x_i, x_j = \begin{cases} 1 & \text{w.p. } \sqrt{x_i x_j} \\ 0 & \text{w.p. } 1 - \sqrt{x_i x_j}. \end{cases}$$

Let Z_d denote the number of non-condom contacts of a node with degree d. If we observe an individual i with d contacts, the probability we search for is $P(Z_d = k)$. To get an expression for $P(Z_d = k)$, we first condition on the non-condom disposition of the sampled individual and then on the non-condom dispositions of the d individuals that the sampled individual met. To be able to distinguish between the sampled individual and his/her partners we will use X_i for our sampled individual and X_j for its partners, but both are of the same distribution.

$$P(Z_d = k) = \int_0^1 P(Z_d = k | X_i = x_i) f_{X_i}(x_i) dx_i$$

$$= \binom{d}{k} \int_0^1 f_{X_i}(x_i) \overbrace{\int_0^1 \cdots \int_0^1}^{k} \overbrace{\int_0^1 \cdots \int_0^1}^{d-k} \sqrt{x_i x_{j_1}} f_{X_j}(x_{j_1}) \cdots \sqrt{x_i x_{j_k}} f_{X_j}(x_{j_k})$$

$$\times \left(1 - \sqrt{x_i x_{j_{k+1}}}\right) f_{X_j}(x_{j_{k+1}}) \cdots \left(1 - \sqrt{x_i x_{j_d}}\right) f_{X_j}(x_{j_d}) dx_{j_1} \cdots dx_{j_d} dx_i$$

$$= \binom{d}{k} \sum_{m=0}^{d-k} (-1)^m \binom{d-k}{m} E\left[X_i^{(k+m)/2}\right] E\left[X_j^{1/2}\right]^{k+m}. \quad \text{(S1.1)}$$

where $E[X]$ is the expected value of the random variable X. The likelihood will be the product of every node's probability given by Eq (S1.1).

We will use two different distributions for the random variable X which we will describe in the following two sections.

S1.1.1 Pro-con model

In this first model an individual's disposition is 0 or 1. When considering the non-condom disposition, either the individual does not want to use a condom (1) or, being pro-condom (0), does. With the geometric mean rule, the following mechanism arises: if an individual who wants to use a condom has sex with an individual who does not they will use a condom. The only way for a condom not to be used is if two individuals with disposition 1 meet.

Let p_N denote the probability that a randomly chosen individual in the population has disposition 1, a non-condom individual. The probability of being pro-condom is $1 - p_N$. Since $X \sim Bern(p_N)$, $E\left[X^d\right] = p_N$ for all d. Using Eq (S1.1) we get

$$P(Z_d = 0) = p_N(1 - p_N)^d + (1 - p_N)$$

$$P(Z_d = k) = p_N \binom{d}{k} p_N^k (1 - p_N)^{d-k} \quad k = 1, \ldots, d.$$

It is assumed that a condom will be used if a condom person meets a non-condom person. This assumption may be relaxed, resulting in an extension of the pro-con model. We add an additional parameter ε_{CN} to govern the probability of non-condom use when a condom person i meets a non-condom person j,

$$P(\text{no condom } | x_i = 0, x_j = 1) = \varepsilon_{CN}.$$

At this stage, when gender is not taken into account, we assume that

$$P(\text{no condom } | x_i = 0, x_j = 1) = \varepsilon_{CN} = \varepsilon_{NC} = P(\text{no condom } | x_i = 1, x_j = 0).$$

In Table S1.1 we have summarised the different non-condom probabilities for the pro-con model.

The case when ε_{CN} is estimated to 0 corresponds to the geometric mean rule for the non-condom probability between a condom individual and a non-condom individual. Note that if $\varepsilon_{CN} > 0$ we have a different rule than the geometric mean rule and the expression for $P(Z_d = k)$ does not hold. Therefore a different method for deriving the likelihood is necessary (see S1.2).

Individual	Condom	Non-condom
Condom	$\varepsilon_{CC} = 0$	ε_{CN}
Non-condom	ε_{NC}	$\varepsilon_{NN} = 1$

Table S1.1: Non-condom probability given the different combinations of types of meeting individuals. ε_{CN} and ε_{NC} stands for the non-condom probability when a condom person meets a non-condom person, ε_{CC} stands for the non-condom probability when two condom persons meet and ε_{NN} when two non-condom persons meet. If $\varepsilon_{CN} = 0$ we have the pro-con model with the geometric mean rule for the non-condom dispositions.

S1.1.2 Continuous model

A suitable continuous choice for the disposition distribution, X, is the Beta distribution since it is quite flexible and has its support on $[0, 1]$. Assume that $X \sim Beta(\alpha, \beta)$, then

$$f_X(x) = \frac{1}{B(\alpha,\beta)} x^{\alpha-1}(1-x)^{\beta-1}, \quad 0 \le x \le 1.$$

See Fig S1.1 for examples of the probability density function of the beta distribution.

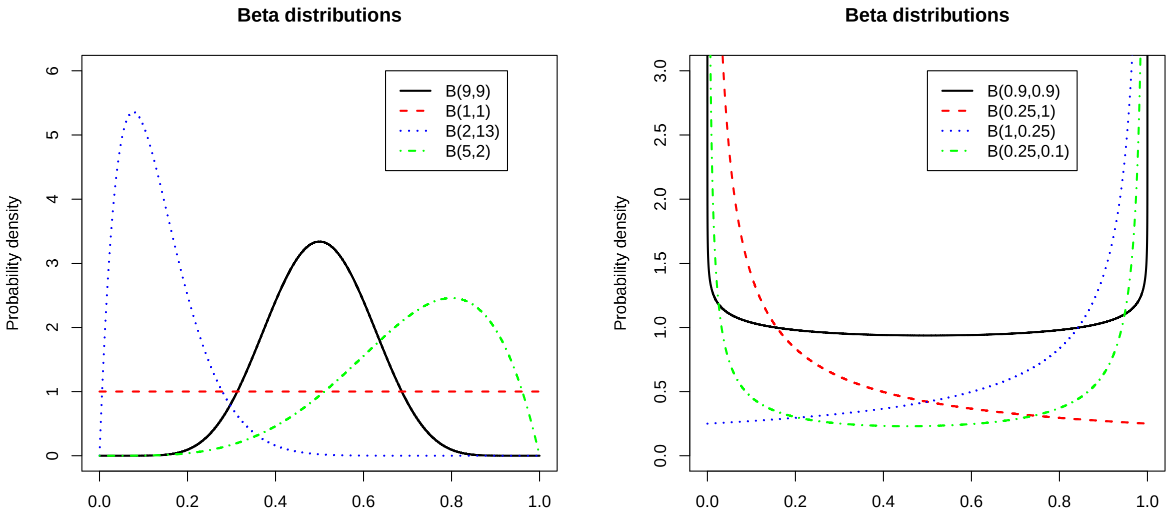

(a) (b)

Fig S1.1: **Different Beta distributions**. Here we give different examples how the beta distribution can look like. The first parameter is corresponding to α and the second one to β

If $X \sim Beta(\alpha, \beta)$ we then also know that

$$E\left[X^k\right] = \frac{\Gamma(\alpha+\beta)\Gamma(\alpha+k)}{\Gamma(\alpha)\Gamma(\alpha+\beta+k)}, \quad k \ge 0.$$

With this information it is possible to get an explicit expression for the probability for an individual to have k non-condom contacts among d sexual contacts

$$P(Z_d = k) = \binom{d}{k} \sum_{m=0}^{d-k} (-1)^m \binom{d-k}{m} \frac{\Gamma(\alpha+\beta)\Gamma(\alpha+\frac{k+m}{2})}{\Gamma(\alpha)\Gamma(\alpha+\beta+\frac{k+m}{2})} \left(\frac{\Gamma(\alpha+\beta)\Gamma(\alpha+1/2)}{\Gamma(\alpha)\Gamma(\alpha+\beta+1/2)}\right)^{k+m}.$$

S1.2 Pro-con-neutral model

The other assumption of translation from the dispositions of two meeting individuals into a probability of condom use is the pro-con-neutral model.

We want to derive the probability of observing an individual with k non-condom contacts among d partners when individuals can be one of three types, a condom individual (C), an indifferent neutral individual (I) or a non-condom individual (N). We also assume that an neutral individual meeting any of the other two categories will do as their partner want. Assume X is a random variable with three possible outcomes C, I and N where $P(X = k) = p_k$ for $k = C, I, N$. The aim is to infer p_C, p_I and p_N, where $p_C + p_I + p_N = 1$. Since the sum of all the three probabilities is 1 it is enough to infer two of them. To this end the probability for one node with d edges to have k non-condom contacts and $d-k$ condom contacts will be determined for arbitrary $0 \leq \varepsilon_{ij} \leq 1$ where $i, j = C, I, N$. As before, let Z_d denote the number of non-condom contacts of a node with degree d. If we condition on what type the studied node is we get

$$P(Z_d = k|p_C, p_N) = \sum_U P(X = U)P(Z_d = k|p_C, p_N, X = U)$$

$$= p_C P(Z_d = k|p_C, p_N, X = C) + p_I P(Z_d = k|p_C, p_N, X = I) + p_N P(Z_d = k|p_C, p_N, X = N). \quad \text{(S1.2)}$$

If a node has k non-condom contacts and $d-k$ condom contacts and $U = C, I, N$, then

$$P(Z_d = k|p_C, p_N, X = U)$$

$$= \binom{d}{k} P(k \text{ non-condom contacts}|p_C, p_N, X = U)P(d-k \text{ condom contacts}|p_C, p_N, X = U). \quad \text{(S1.3)}$$

In order to obtain an expression for $P(Z_d = k|p_C, p_N, X = U)$ where $U = C, I, N$ we need to condition on how many of the partners is of each type, C, I or N. We have that

$$P(k \text{ non-condom contacts}|p_C, p_N, X = U)$$

$$= \sum_{m=0}^{k} \binom{k}{m} p_C^m \sum_{i=0}^{k-m} \binom{k-m}{i} p_I^i p_N^{k-m-i} \varepsilon_{UC}^m \varepsilon_{UI}^i \varepsilon_{UN}^{k-m-i}$$

$$P(k \text{ condom contacts}|p_C, p_N, X = U)$$

$$= \sum_{m=0}^{k} \binom{k}{m} p_C^m \sum_{i=0}^{k-m} \binom{k-m}{i} p_I^i p_N^{k-m-i} (1-\varepsilon_{UC})^m (1-\varepsilon_{UI})^i (1-\varepsilon_{UN})^{k-m-i}.$$

To create the model where neutral individuals will do as condom or non-condom individuals want, we fix ε_{ij} according to Table S1.2.

S1.3 Gender asymmetries

Both main models, the continuous and pro-con-neutral model, can be extended to take gender (here men and women) into account. In Eq S1.1 indices were kept even if they were not necessary. This was done in order to be able to separate from which individual each contribution in the probability came from, the sampled individual or its partners. Therefore, in a heterosexual network, we can easily separate the likelihood into a male part and a female part, if we assume that male dispositions are independent of female dispositions but that they may differ in distribution between genders. Let Z_d^W denote the number of non-condom contacts

	Condom	Neutral	Non-condom
Condom	$\varepsilon_{CC} = 0$	$\varepsilon_{CI} = 0$	ε_{CN}
Neutral	$\varepsilon_{IC} = 0$	$\varepsilon_{II} = 0.5$	$\varepsilon_{IN} = 1$
Non-condom	ε_{NC}	$\varepsilon_{NI} = 1$	$\varepsilon_{NN} = 1$

Table S1.2: Non-condom probability given the different combinations of types of meeting individuals. A condom individual is denoted by C, a neutral by I and a non-condom individual by N. ε_{ij} where $i, j = C, I, N$ is the non-condom probability when a person of type i meets a person of type j.

of a woman with degree d. Let Z_d^M be defined in the same way but for men. For the model where the non-condom probability of two individuals who have sex will be given by the geometric mean of the two individuals' dispositions we have

$$P(Z_d^W = k) = \binom{d}{k} \sum_{m=0}^{d-k} (-1)^m \binom{d-k}{m} E\left[X_W^{(k+m)/2}\right] E\left[X_M^{1/2}\right]^{k+m}$$

$$P(Z_d^M = k) = \binom{d}{k} \sum_{m=0}^{d-k} (-1)^m \binom{d-k}{m} E\left[X_M^{(k+m)/2}\right] E\left[X_W^{1/2}\right]^{k+m}.$$

It is also possible to model and infer if females (or males) are the one who's opinion weigh higher. Let x_M be the non-condom disposition of a man meeting a woman with non-condom disposition x_W. We extend the previous model by introducing a new parameter γ

$$Y_{MF}|x_M, x_F = \begin{cases} 1 & \text{w.p. } x_M^{\gamma} x_F^{1-\gamma} \\ 0 & \text{w.p. } 1 - x_M^{\gamma} x_F^{1-\gamma} \end{cases}$$

If γ is 0.5 then the geometric mean is the rule which determine the probability of non-condom use. If $\gamma = 1$ then only the male disposition matters, if $\gamma = 0$ only the female disposition matter. If $0.5 < \gamma \leq 1$ then the male disposition weigh higher, since $x_M^{\gamma} x_F^{1-\gamma}$ is shifted towards X_M in comparison to $\sqrt{x_M x_F}$. If $0 \leq \gamma < 0.5$ then the female disposition weigh higher, since $x_M^{\gamma} x_F^{1-\gamma}$ is shifted towards X_F in comparison to $\sqrt{x_M x_F}$.

For the pro-con-neutral disposition model it is also simple to incorporate gender if we study heterosexual contacts. Assume that women draw their disposition from a distribution where being for condom, C^W, happens with a probability p_C^W, being neutral happens with a probability p_I^W and being against condom happens with a probability p_N^W. For men the corresponding probabilities are p_C^M, p_I^M and p_N^M. If we want to determine the probability that a woman has k non-condom contacts among d contacts we can use a modified version of Eq (S1.2) where we will have

$$P(Z_d^W = k | p_C^W, p_N^W, p_C^M, p_N^M)$$

$$= p_C^W P(Z_d^W = k | p_C^M, p_N^M, X = C) + p_I^W P(Z_d^W = k | p_C^M, p_N^M, X = I) + p_N^W P(Z_d^W = k | p_C^M, p_N^M, X = N).$$

For each probability $P(Z_d^W = k | p_C^M, p_N^M, X = U)$, where $U = C, I, N$, we can use Eq (S1.3). In the same manner we get $P(Z_d^M = k | p_C^W, p_N^W, p_C^M, p_N^M)$.

S1.4 Different types of sexual relationships

An individual has two dispositions (x_1, x_2), one for each relationship type. We want to test the null hyopthesis that individuals use the same disposition in the two relationship types, that $x_1 = x_2$, or if $x_1 < x_2$. This is

achieved by constructing a model with an additional parameter q to represent these hypotheses. If individuals use the same disposition in both relationship types we will have that

$$H_0 : q = 0,$$

and if that is not the case

$$H_1 : q > 0.$$

To achieve this, let Z, Z_1, Z_2 be independent random variables with the same distribution F with parameters $\boldsymbol{\theta}$.
Let $Z_{(1)} = \min(Z_1, Z_2)$ and $Z_{(2)} = \max(Z_1, Z_2)$. Now form new variables X_1 and X_2 by introducing a parameter q which represents the probability for an individual to draw its dispositions from the ordered sample

$$\begin{aligned} X_1 = X_2 = Z \quad &\text{w.p. } 1-q \\ X_1 = Z_{(1)} \text{ and } X_2 = Z_{(2)} \quad &\text{w.p. } q \end{aligned}$$

The parameter q will in this model lie between 0 and 1. H_0 corresponds to $q = 0$, since then $X_1 = X_2$. If $q = 1$, X_1 is the minimum of Z_1 and Z_2, and X_2 the maximum of Z_1 and Z_2. If $0 < q < 1$, X_1 and X_2 could be equal for some individuals. In the analysis we will assume two different distributions F. In one case F will be a Beta distribution with parameters $\boldsymbol{\theta} = (\alpha, \beta)$. In the other we will assume the pro-con-neutral disposition model where F is governed by the parameters $\boldsymbol{\theta} = (p_C, p_I, p_N)$. Our aim is then to infer these parameters of the models as well as the probability q in order to see if we can reject our null hypothesis (corresponding to $q = 0$) or not.

There will be some differences in the two cases, since the Beta distribution is continuous and in the pro-con-neutral disposition model F will be discrete. In the former case

$$\begin{aligned} X_1 = X_2 \quad &\text{w.p. } 1-q \\ X_1 < X_2 \quad &\text{w.p. } q \end{aligned}$$

In the latter case, given that $q > 0$, it is still possible that $Z_{(1)} = Z_{(2)}$ since Z_1 and Z_2 are discrete random variables. This happens with probability $p_C^2 + p_I^2 + p_N^2$, and therefore

$$X_1 < X_2 \quad \text{w.p. } q\left(1 - \left(p_C^2 + p_I^2 + p_N^2\right)\right)$$

The likelihood for this model (for which we can assign different $Z \sim F$) will be given by the product of the probability, for each individual, of having m_1 non-condom contacts among d_1 contacts in total in relationship type 1, and having m_2 non-condom contacts among d_2 contacts in total in relationship type 2. If we are interested in this probability for individual i we need to condition whether this individual's dispositions is of the ordered sample or if they are the same in the two relationship types.

$$\begin{aligned} P(m_1, m_2 | q, \boldsymbol{\theta}) =& (1-q) P(m_1, m_2 | q, \boldsymbol{\theta}, X_1 = Z, X_2 = Z) \\ &+ qP\left(m_1, m_2 | q, \boldsymbol{\theta}, X_1 = Z_{(1)}, X_2 = Z_{(2)}\right) \end{aligned} \tag{S1.4}$$

Eq (S1.4) will depend on what distribution we choose for F, the non-condom disposition distribution. We seek the probability that an individual has m_1 number of non-condom contacts of type I conditioned on the total number of contacts of type I, m_2 non-condom contacts of type II conditioned on the total number of contacts of type II.

S1.5 Gender asymmetries in the Continuous model

Let us first assume that F is a Beta distribution with parameters $\boldsymbol{\theta} = (\alpha, \beta)$, our aim is to infer these parameters as well as the probability q.
When we condition on that the dispositions of the studied individual is the same in both relationship types, we get

$$P(m_1, m_2|q, \boldsymbol{\theta}, X_1 = Z, X_2 = Z) = P(m_1, m_2|q, \boldsymbol{\theta}, X_1 = X_2)$$

$$= \int_0^1 P(m_1|\boldsymbol{\theta}, X_1 = x)P(m_1|\boldsymbol{\theta}, X_2 = x)f_Z(x)dx$$

$$= \int_0^1 \binom{d_1}{m_1} \left(\sqrt{x}E\left[\sqrt{X_1}\right]\right)^{m_1} \left(1 - \sqrt{x}E\left[\sqrt{X_1}\right]\right)^{d_1 - m_1}$$

$$\times \binom{d_2}{m_2} \left(\sqrt{x}E\left[\sqrt{X_2}\right]\right)^{m_2} \left(1 - \sqrt{x}E\left[\sqrt{X_2}\right]\right)^{d_2 - m_2} f_Z(x)dx$$

But now we do also need to condition on whether the partners of individual i has the same disposition or not. For partners in relationship type 1 we will have that

$$E\left[\sqrt{X_1}\right] = (1-q)E\left[\sqrt{Z}\right] + qE\left[\sqrt{Z_{(1)}}\right]$$

For partners in relationship type 2 we will have

$$E\left[\sqrt{X_2}\right] = (1-q)E\left[\sqrt{Z}\right] + qE\left[\sqrt{Z_{(2)}}\right]$$

Now let us examine the second part of Eq (S1.4), we need the distribution of the order statistic. If $(Z_1, ..., Z_n)$ are exchangeable, with joint p.d.f. $f_{Z_1,...,Z_n}$, the joint p.d.f. of the ordered observations $X_i = Z_{(i)}$ is

$$f_{X_1,...,X_n}(q_1, ..., q_n) = n! f_{Z_1,...,Z_n}(q_1, ..., q_n)$$

if $q_1 < q_2 < ... < q_n$, and 0 otherwise. Note that this is for continuous distributions F.

Conditioned on that $X_1 = Z_{(1)}$ and $X_2 = Z_{(2)}$ we know that $X_1 < X_2$. Again, let us condition on the disposition in relationship type 1 and the disposition in relationship type 2 of our studied individual, now knowing they are different and ordered.

$$P(m_1, m_2|q, \boldsymbol{\theta}, X_1 = Z_{(1)}, X_2 = Z_{(2)})$$

$$= P(m_1, m_2|q, \boldsymbol{\theta}, X_1 < X_2)$$

$$= \int_0^1 \int_0^1 P(m_1, m_2|q, \boldsymbol{\theta}, X_1 < X_2, x_1, x_2) f_{X_1,X_2}(x_1, x_2|X_1 < X_2, \boldsymbol{\theta}) dx_2 dx_1$$

$$= \int_0^1 \int_{x_1}^1 P(m_1, m_2|q, \boldsymbol{\theta}, X_1 < X_2, x_1, x_2) f_{X_1,X_2}(x_1, x_2|X_1 < X_2, \boldsymbol{\theta}) dx_2 dx_1$$

$$= \int_0^1 \int_{x_1}^1 P(m_1|q, \boldsymbol{\theta}, x_1) P(m_2|q, \boldsymbol{\theta}, x_2) f_{X_1,X_2}(x_1, x_2|X_1 < X_2, \boldsymbol{\theta}) dx_2 dx_1$$

$$= \int_0^1 \int_{x_1}^1 \left(\prod_{i=1}^{2} \binom{d_i}{m_i} \left(\sqrt{x_i} E\left[\sqrt{X_i}\right]\right)^{m_i} \left(1 - \sqrt{x_i} E\left[\sqrt{X_i}\right]\right)^{d_i - m_i} \right) f_{X_1,X_2}(x_1, x_2|X_1 < X_2, \boldsymbol{\theta}) dx_2 dx_1$$

$$= 2! \int_0^1 \int_{x_1}^1 \prod_{i=1}^{2} \binom{d_i}{m_i} \left(\sqrt{x_i} E\left[\sqrt{X_i}\right]\right)^{m_i} \left(1 - \sqrt{x_i} E\left[\sqrt{X_i}\right]\right)^{d_i - m_i} f_{Z_1}(x_1|\boldsymbol{\theta}) f_{Z_2}(x_2|\boldsymbol{\theta}) dx_2 dx_1$$

Note that

$$E\left[\sqrt{X_i}\right] = (1-q)E\left[\sqrt{Z}\right] + qE\left[\sqrt{Z_{(i)}}\right] \quad i = 1, 2$$

We have not found a closed form of the probability conditioned on $X_1 < X_2$. To do inference we have calculated this probability with numerical integration.

S1.5.1 Gender and different relationship types

The above model which takes two different relationship types into account can be extended to also incorporate gender. We assume that there are two different underlying distributions, F^W for women and F^M for men. Also, assume that women and men have different probabilities q^w and q^m to draw their dispositions from the ordered sample. The contribution to the likelihood from men and women will then differ. Assume that we want to calculate the contribution from a woman with d_1^W partners in relationship type 1 and d_2^W partners in relationship type 2. If we want to calculate the probability that this woman has m_1^W non-condom contacts in relationship type 1 and m_2^W non-condom contacts in relationship type 2, then we start by conditioning if this woman draw her dispositions from the same distribution or from the ordered sample.

$$P(m_1^W, m_2^W|q^W, q^M, \boldsymbol{\theta})$$

$$= P(m_1^W, m_2^W|q^M, \boldsymbol{\theta}, X_1 = X_2)(1 - q^W) + P\left(m_1^W, m_2^W|q^M, \boldsymbol{\theta}, X_1 < X_2\right) q^W \tag{S1.5}$$

Conditioned on that this women use the same disposition in both relationship types we get

$$P(m_1^W, m_2^W | q^M, \boldsymbol{\theta}, X_1 = X_2) = \int_0^1 \binom{d_1^W}{m_1^W} \left(\sqrt{x} E\left[\sqrt{X_1^M}\right]\right)^{m_1^W} \left(1 - \sqrt{x} E\left[\sqrt{X_1^M}\right]\right)^{d_1^W - m_1^W}$$

$$\times \binom{d_2^W}{m_2^W} \left(\sqrt{x} E\left[\sqrt{X_2^M}\right]\right)^{m_2^W} \left(1 - \sqrt{x} E\left[\sqrt{X_2^M}\right]\right)^{d_2^W - m_2^W} f_Z^W(x) dx$$

where

$$E\left[\sqrt{X_i^M}\right] = (1 - q^M) E\left[\sqrt{Z^M}\right] + q^M E\left[\sqrt{Z_{(i)}^M}\right] \quad i = 1, 2$$

Conditioned on that this woman use dispositions from an order sample we get

$$P(m_1^W, m_2^W | q^W, q^M, \boldsymbol{\theta}, X_1 < X_2)$$

$$= 2! \int_0^1 \int_{x_1}^1 \prod_{i=1}^{2} \binom{n_i^W}{m_i^W} \left(\sqrt{x_i} E\left[\sqrt{X_i^M}\right]\right)^{m_i^W} \left(1 - \sqrt{x_i} E\left[\sqrt{X_i^M}\right]\right)^{d_i^W - m_i^W} f_{Z_1^W}(x_1 | \boldsymbol{\theta}) f_{Z_2^W}(x_2 | \boldsymbol{\theta}) dx_2 dx_1$$

To find $P(m_1^M, m_2^M | q^W, q^M, \boldsymbol{\theta})$ we proceed in the same way as for women.

S1.6 Gender asymmetries in thr Pro-con-neutral disposition model

Now the same kind of extension will be done for the model where extreme opinion weigh higher and with three categories of individuals. Assume that individuals draw their disposition for a casual type of relationship from a random variable X_1 and its disposition for a steady and regular relationship from a random variable X_2, both with the three outcomes C (0), I (0.5) and N (1). The null hypothesis is that $X_1 = X_2$ and the alternative hypothesis that $X_1 < X_2$ which corresponds to that people in casual relationship tend to use condom more often.

Let Z be a random variable that have three outcomes, where $P(Z = C) = p_C$, $P(Z = I) = p_I$ and $P(Z = N) = p_N$. Let Z_1 and Z_2 be two random variables which have the same distribution as Z, that are independent of each other and of Z. As before, let $Z_{(1)} = \min(Z_1, Z_2)$ and $Z_{(2)} = \max(Z_1, Z_2)$ and

$$X_1 = Z \text{ and } X_2 = Z \quad \text{w.p. } 1 - q$$
$$X_1 = Z_{(1)} \text{ and } X_2 = Z_{(2)} \quad \text{w.p. } q$$

Again our H_0 corresponds to $q = 0$. If $q = 1$, X_1 and X_2 are the ordered sample of Z_1 and Z_2. Let m_i denote the number of non-condom contacts and in relationship type i, $i = 1, 2$, d_i is defined in the same manner but for the total number of contacts. The total number of contacts of each relationship type is assumed to be known. The likelihood for this model, where extreme opinion weighs higher, will be given by the product of each nodes probability. The parameters to infer additional to q are $\boldsymbol{\theta} = (p, p_C, p_N)$, $(p_I = 1 - p_C - p_N)$. Eq (S1.4) will now become

$$P(m_1, m_2 | q, \boldsymbol{\theta}) = (1 - q) P(m_1, m_2 | q, p_C, p_N, X_1 = Z, X_2 = Z)$$

$$+ q P\left(m_1, m_2 | q, p_C, p_N, X_1 = Z_{(1)}, X_2 = Z_{(2)}\right) \quad \text{(S1.6)}$$

With probability $1-q$ the random variables X_1 and X_2 will be equal to the same random variable Z. With probability q we will have that $X_1 = X_{(1)}$ and $X_2 = X_{(2)}$. In the latter case it is still possible that $X_{(1)} = X_{(2)}$ since Z_1 and Z_2 are discrete random variables, this happens with probability $p_C^2 + p_I^2 + p_N^2$. Let $q' = q(1-(p_C^2+p_I^2+p_N^2))$ therefore we have that

$$\begin{aligned} X_1 = X_2 &\quad \text{w.p. } 1-q' \\ X_1 < X_2 &\quad \text{w.p. } q'. \end{aligned}$$

Let $k = C, I, N$. To get the first probability in Eq (S1.6), $P(m_1, m_2|q, p_C, p_N, X_1 = Z, X_2 = Z)$, we assume $(X_1 = Z, X_2 = Z)$. The probability of observing m_1 non-condom contacts in relationship type 1 and m_2 non-condom contacts in relationship type 2 is then given by

$$P(m_1, m_2|q, p_C, p_N, X_1 = Z, X_2 = Z) = \sum_k P(m_1, m_2|q, p_C, p_N, X_1 = k, X_2 = k)P(Z = k)$$

$$= \sum_k P(m_1|q, p_C, p_N, X_1 = k)P(m_2|q, p_C, p_N, X_2 = k)P(Z = k) \qquad \text{(S1.7)}$$

We can use $P(Z_d = k|p_C, p_N, X = U)$, $U = C, I, N$, from Section 2.1.4 with some modifications in order to determine $P(m_1|q, p_C, p_N, X_1 = k)$ and $P(m_2|q, p_C, p_N, X_1 = k)$. The parts needs changing are the probabilities that a partner is of a certain type, $P(X = C) = p_C$, $P(X = I) = p_I$ and $P(X = N) = p_N$. We need to condition on whether the partners draw their dispositions in the two different relationship types from the same distribution or from the ordered sample. If all partners would draw their dispositions from the same distribution we could have used $P(Z_d = k|p_C, p_N, X = U)$ from Section 2.1.4 without modification, because then $P(X_1 = I) = P(X_2 = I) = p_I$, $P(X_1 = C) = P(X_2 = C) = p_C$ and $P(X_1 = N) = P(X_2 = N) = p_N$. For one partner $X_1 = Z$ and $X_2 = Z$ with probability $1-q$, then $P(X_1 = I) = P(X_2 = I) = p_I$, $P(X_1 = C) = P(X_2 = C) = p_C$ and $P(X_1 = N) = P(X_2 = N) = p_N$ for this partner. If the partner draw its dispositions from an ordered sample it gets a little more complicated. The probability that a partner is a condom person, that it has disposition C, when it draws it disposition from an ordered sample is the same as disposition C being the minimum of Z_1 and Z_2, this is

$$P(Z_{(1)} = C) = p_C(p_C + p_I + p_N) + p_C(p_I + p_N)$$

However, the probability that one partner has the disposition C in relationship 1, $P(X_1 = C)$, is

$$P(X_1 = C) = P(X_1 = C|X_1 = X_2 = Z)(1-q) + P(X_1 = C|X_1 = Z_{(1)}, X_2 = Z_{(2)})q$$

$$= P(Z = C)(1-q) + P(Z_{(1)} = C)q$$

$$= p_C(1-q) + (p_C + p_C(p_I + p_N))q$$

$$= p_C(1-q) + (2p_C - p_C^2)q$$

The second part of this probability comes from the probability of observing the minimum of Z_1 and Z_2 to be C. For the other two possible dispositions in relationship type 1 we have

$$\begin{aligned} P(X_1 = I) &= p_I(1-q) + (p_I^2 + 2p_I p_N)q \\ P(X_1 = N) &= p_N(1-q) + p_N^2 q \end{aligned}$$

If we instead are interested in the probability to observe different dispositions in partners in relationship type 2 we for example have that the probability that a partner is of type C is

$$P(X_2 = C) = P(X_2 = C|X_1 = X_2 = Z)(1-q) + P(X_1 = C|X_1 = Z_{(1)}, X_2 = Z_{(2)})q$$

$$= P(Z = C)(1-q) + P(Z_{(2)} = C)q$$

$$= p_C(1-q) + p_C^2 q$$

For the other two possible dispositions in relationship type 2 we have

$$P(X_2 = I) = p_I(1-q) + (p_I^2 + 2p_I p_C)q$$
$$P(X_2 = N) = p_N(1-q) + (2p_N - p_N^2)q$$

We now have all needed for the probability $P(m_1, m_2|q, p_C, p_N, X_1 = Z, X_2 = Z)$ in Eq (S1.6).

To get the second probability, $P\left(m_1, m_2|q, p_C, p_N, \{X_1 = Z, X_2 = Z\}^C\right)$, in Eq (S1.6) we will also need these $P(X_i = C)$, $P(X_i = I)$ and $P(X_i = N)$ for $i = 1, 2$. Now assume that it is known that $\{X_1 = Z, X_2 = Z\}^C = \{X_1 = Z_{(1)}, X_2 = Z_{(2)}\}$ and let $k_i = C, I, N$, $i = 1, 2$, then

$$P\left(m_1, m_2|q, p_C, p_N, X_1 = Z_{(1)}, X_2 = Z_{(2)}\right) = P(m_1, m_2|q, p_C, p_N, X_1 \leq X_2)$$

$$= \sum_{k_2=0}^{1} \sum_{k_1=0}^{1} P(m_1|q, p_C, p_N, X_1 \leq X_2, X_1 = k_1) P(m_2|q, p_C, p_N, X_1 \leq X_2, X_2 = k_2) P(X_1 = k_1, X_2 = k_2)$$

$$= \sum_{k_2=0}^{1} \sum_{k_1 \leq k_2} P(m_1|q, p_C, p_N, X_1 = k_1) P(m_2|q, p_C, p_N, X_2 = k_2) P(X_1 = k_1, X_2 = k_2)$$

Again, we may use $P(Z_d = k|p_C, p_N, X = U)$, $U = C, I, N$, from Section 2.1.4 with the same modification for the probabilities of a partner to be a certain type. Also, the probability $P(X_1 = k_1, X_2 = k_2)$ is given by

$$P(X_1 = k_1, X_2 = k_2) = \begin{cases} 2P(Y_1 = k_1)P(Y_2 = k_2) & = 2p_{k_1}p_{k_2} \quad \text{if } k_1 < k_2 \\ P(Y_1 = k_1)^2 & = p_{k_1}^2 \quad \text{if } k_1 = k_2 \end{cases}$$

S1.6.1 Different types of relationship and gender

It also here simple to incorporate gender into the model which takes two relationship into account. Let Z^W and Z^M be two random variables with three outcomes each. The probabilities for the different types for women are p_C^W, p_I^W, p_N^W and for men p_C^M, p_I^M, p_N^M. As in the previous section we assume that individuals may draw their dispositions from an ordered sample with a probability which we want to infer. For women this probability is q^W and for men q^M. We do this by constructing the random variables X_1^W and X_2^W in the following way

$$X_1^W = Z^W \text{ and } X_2^W = Z^W \quad \text{w.p. } 1 - q^W$$
$$X_1^W = Z_{(1)}^W \text{ and } X_2^W = Z_{(2)}^W \quad \text{w.p. } q^W$$

For men X_1^M and X_2^M are defined in the same manner.
Let us calculate the probability that a woman has m_1 non-condom contacts in relationship type 1 among d_1 contacts in relationship type 1, and m_2 non-condom contacts in relationship type 2 among d_2 contacts in relationship type 2. If $\boldsymbol{\theta} = (p_C^W, p_N^W, p^W, p_C^M, p_N^M)$ then

$$
\begin{aligned}
&P(m_1, m_2|q^W, q^M, \boldsymbol{\theta})\\
=&(1-q^W)P(m_1, m_2|q^M, \boldsymbol{\theta}, X_1^W = Z^W, X_2^W = Z^W) + q^W P\left(m_1, m_2|q^M, \boldsymbol{\theta}, \{X_1^W = Z^W, X_2^W = Z^W\}^C\right)
\end{aligned}
$$

If $k = C, I, N$, then

$$
\begin{aligned}
&P(m_1, m_2|q^M, \boldsymbol{\theta}, X_1^W = Z^W, X_2^W = Z^W)\\
&= \sum_k P(m_1|q^M, p_C^M, p_N^M, X_1^W = k)P(m_2|q^M, p_C^M, p_N^M, X_2^W = k)P(Z^W = k)
\end{aligned}
$$
