# Supplementary material for: Inferring individual sexual action dispositions from egocentric network data on dyadic sexual outcomes
Source: PLoS One. 2018 Nov 12;13(11):e0207116. doi: 10.1371/journal.pone.0207116 (PMC6231623; doi:10.1371/journal.pone.0207116)
Supplement: S2 Appendix — (PDF) [file pone.0207116.s002.pdf]

Appendix S2: Simulation Results

In order to verify how well the models manage to give reliable estimates we did a simulation study. First, we simulate a network with the same degree distribution as the real-world data set with a configuration model (explained below). Then we give each node in the network a disposition, which is used to determine whether a condom was used or not in the different relationships. The disposition given and the rule to determine if a condom is used will be different depending on which model we are testing. Lastly, from the simulated sexual network we extract what will be to us the visible data: the types of the edges but removed the information of what type each node has and who has a connection to who. On this simulated data the Likelihoods from the Section *Model of disposition to sexual outcome* are tested (ML approach).

A short explanation of how the network was simulated is as follows: assume we have n females with degree distribution $\{d_1, \ldots, d_n\}$ and m males with degree distribution $\{d_{n+1}, \ldots, d_{n+m}\}$. Each individual $i \in [1, \ldots, n+m]$ is seen as having d_i half-stubs. We then randomly choose one stub of the females and randomly choose one stub of the males and connect them. Of the free stubs we continue to take randomly one stub of the females and one of the males and connect them until we run out of free stubs from any of the genders.

In the true underlying sexual network there are almost as many men as females. Therefore, when simulating the networks we use 448 men instead of the sample size of men of 224. This is also needed when using the configuration model to create simulated networks from which we are able to extract a sample of 422 women from. If we only used 224 men we would run out of male stubs too fast in order to create a network where we had over 400 females with at least one partner. When analysis is done we do however randomly choose 224 of the males to include in our simulated sample.

S2.1 Pro-con model

We simulated a network with 646 nodes according to the degree distribution of the real data. On this type of network, a fraction of p_N was chosen to be non-condom individuals and the rest was assigned as a condom individual. The edges between nodes was assigned its type according to the rule given in the main text.

Table S2.1 summarises the result of 100 simulations. From Table S2.1 we see that if the true model generating the observed data, was indeed the pro-con model we would get the correct estimates.

true p_N	mean $\hat{p}_N$	s.d $\hat{p}_N$
0.9	0.898	0.012
0.7	0.696	0.018
0.5	0.492	0.024
0.3	0.298	0.024
0.1	0.096	0.019

Table S2.1: Simulated configuration network with the degree distribution of the real-world data set. The rule for condom use follows the pro-con model where the parameter ε_{CN} is fixed to 0. The sample size drawn from the simulated network is $n = 646$. We did 100 simulations for each set-up and estimated the fraction of non-condom persons p_N for each simulated sample. Note that we here show the means and the standard deviation of the 100 estimates.

We also simulate the pro-con model with the additional parameter ε_{CN}. This parameter governs the probability of not using a condom when a condom person meets a non-condom person. Table S2.2 shows the summary of the estimates. We also tested if we would be able to reject the null hypothesis that $\varepsilon_{CN} = 0$ for each of the simulated networks. In Table S2.3 we see that we almost every time reject the null model (likelihood ratio test) in favour of the model assuming that $\varepsilon_{CN} > 0$ on the 5% level.

The exceptions are when the dispositions are distributed in a manner close to the null model: when the condom persons are many and favours the condom persons disposition, i.e. when ε_{CN} is close to 0. For example, when the condom persons are of 85% and when $\varepsilon_{CN} = 0.1$. In this scenario the majority are

condom persons and the probability of using a condom if a non-condom person meets a condom person is 90%. The estimates for the pro-con model with the parameter ε_{CN} as a free parameter is able to get good estimates, but when testing the model against the null model where $\varepsilon_{CN} = 0$ we do not get as big difference in likelihood as needed to be able to reject the null model on the 5% level in half the cases. The same goes for when 95% of the individuals in the sampe are condom persons and ε_{CN} is 0.1 or 0.3.

True values		Mean and s.d. of estimates			
p_N	ε_{CN}	$\hat{p}_N$	s.d $\hat{p}_N$	$\hat{\varepsilon}_{CN}$	s.d $\hat{\varepsilon}_{CN}$
	0.1	0.749	0.017	0.100	0.023
	0.3	0.750	0.025	0.299	0.048
0.75	0.5	0.738	0.037	0.524	0.077
	0.7	0.745	0.042	0.708	0.088
	0.9	0.759	0.049	0.879	0.080
	0.1	0.503	0.030	0.097	0.033
	0.3	0.498	0.043	0.303	0.066
0.5	0.5	0.494	0.043	0.509	0.076
	0.7	0.504	0.043	0.694	0.079
	0.9	0.501	0.028	0.901	0.029
	0.1	0.145	0.046	0.127	0.102
	0.3	0.173	0.050	0.272	0.107
0.15	0.5	0.158	0.034	0.487	0.091
	0.7	0.151	0.019	0.707	0.057
	0.9	0.151	0.015	0.902	0.027
	0.1	0.059	0.033	0.138	0.148
	0.3	0.075	0.045	0.245	0.144
0.05	0.5	0.052	0.018	0.505	0.12
	0.7	0.054	0.020	0.693	0.102
	0.9	0.048	0.009	0.899	0.046

Table S2.2: Simulated configuration network with the degree distribution of the real-world data set. The rule for condom use follows the pro-con model with the additional parameter ε_{CN} is allowed to vary. The sample size drawn from the simulated network is $n = 646$. We did 100 simulations for each set-up and estimated the parameter ε_{CN} and the fraction of non-condom persons p_N for each simulated sample. Note that we here show the means and the standard deviation of the 100 estimates.

True values					
p_N	ε_{CN}	$\%p < 0.05$	$\%p < 0.01$	$\%p < 0.005$	$\%p < 0.001$
	0.1	100	100	100	100
	0.3	100	100	100	100
0.75	0.5	100	100	100	100
	0.7	100	100	100	100
	0.9	100	100	100	100
	0.1	100	100	100	97
	0.3	100	100	100	100
0.5	0.5	100	100	100	100
	0.7	100	100	100	100
	0.9	100	100	100	100
	0.1	54	36	30	13
	0.3	100	97	97	91
0.15	0.5	100	100	100	100
	0.7	100	100	100	100
	0.9	100	100	100	100
	0.1	8	0	0	0
	0.3	50	27	21	11
0.05	0.5	97	94	92	86
	0.7	99	99	99	99
	0.9	100	100	100	100

Table S2.3: For the same simulated networks as in Table S2.2 we tested if we could reject the null model assuming $\varepsilon_{CN} = 0$ in favuor of the model where ε_{CN} is a free parameter. This was done with a likelihood-ratio test.

S2.2 Pro-con-neutral model

Next, we simulated data according to the model where strong opinion weigh higher in the decision whether a condom is used. In Table S2.4 the estimates for the simulated data were relationship types or gender is not taken into account are shown.

Additionally, when we study the anal sex dispositions we do at one point only consider the casual sexual relationships, with a sample size of 456 individuals. We did therefore do additional simulations with this sample size and degree distribution, the results are found in S2.5.

True values		Mean and s.d. of estimates			
p_C	p_N	$\hat{p}_C$	s.d $\hat{p}_C$	$\hat{p}_N$	s.d $\hat{p}_N$
0.05	0.9	0.049	0.009	0.907	0.049
	0.5	0.048	0.011	0.500	0.030
	0.25	0.046	0.012	0.246	0.025
	0.1	0.049	0.015	0.103	0.019
0.4	0.5	0.400	0.024	0.497	0.084
	0.25	0.396	0.028	0.248	0.046
	0.1	0.402	0.030	0.112	0.052

Table S2.4: Simulated configuration network with the degree distribution of the real-world data set. Dispositions and actions were set according to the pro-con-neutral model where it is assumed that men and women have the same disposition distribution. The sample size drawn from the simulated network is $n = 646$. We did 100 simulations for each parameter set-up and then estimated the parameters.

True values		Mean and s.d. of estimates			
p_C	p_N	$\hat{p}_C$	s.d $\hat{p}_C$	$\hat{p}_N$	s.d $\hat{p}_N$
0.05	0.9	0.048	0.008	0.885	0.080
	0.5	0.053	0.014	0.512	0.068
	0.25	0.048	0.015	0.251	0.026
	0.1	0.049	0.017	0.100	0.024
0.4	0.5	0.390	0.022	0.491	0.101
	0.3	0.400	0.031	0.310	0.072
	0.1	0.405	0.044	0.119	0.087

Table S2.5: Simulated configuration network with the degree distribution of the real-world data set. Dispositions and actions were set according to the pro-con-neutral model where it is assumed that men and women have the same disposition distribution. The sample size drawn from the simulated network is $n = 456$. We did 100 simulations for each parameter set-up and then estimated the parameters.

We also simulate the pro-con-neutral model with the additional parameter ε_{CN}. Table S2.6 shows the summary of the estimates. We also tested if we would be able to reject the null hypothesis that $\varepsilon_{CN} = 0$ for each of the simulated networks. In Table S2.7 we see that we almost every time reject the null model (likelihood ratio test) on the 5% level. The exception occurs when almost everyone is a non-condom person and a small fraction is a condom person and neutral. Then almost all contacts will be without a condom regardless of the value of ε_{CN}.

True values			Mean and s.d. of estimates					
p_C	p_N	ε_{CN}	$\hat{p}_C$	s.d $\hat{p}_C$	$\hat{p}_N$	s.d $\hat{p}_N$	$\hat{\varepsilon}_{CN}$	sd $\hat{\varepsilon}_{CN}$
		0	0.048	0.008	0.889	0.066	0.001	0.005
0.05	0.9	0.25	0.047	0.012	0.893	0.067	0.230	0.086
		0.5	0.047	0.026	0.879	0.082	0.460	0.158
		0.75	0.048	0.059	0.845	0.072	0.600	0.330
		0.5	0.799	0.093	0.067	0.037	0.518	0.162
0.76	0.06	0.75	0.788	0.084	0.060	0.016	0.762	0.090
		0.9	0.774	0.074	0.061	0.012	0.904	0.064

Table S2.6: Simulated configuration network with the degree distribution of the real-world data set. Dispositions and actions were set according to the pro-con-neutral model with the extra parameter ε_{CN}. It is assumed that men and women have the same disposition distribution. The sample size drawn from the simulated network is $n = 456$. We did 100 simulations for each parameter set-up and then estimated the parameters.

True values						
p_C	p_N	ε_{CN}	$\%p < 0.05$	$\%p < 0.01$	$\%p < 0.005$	$\%p < 0.001$
0.05	0.9	0.25	96	91	86	81
		0.5	90	78	73	57
		0.75	24	15	11	6
		0.5	94	89	87	77
0.76	0.06	0.75	100	99	99	97
		0.9	100	100	100	100

Table S2.7: For the same simulated networks as in Table S2.6 we tested if we could reject the null model assuming $\varepsilon_{CN} = 0$ in favuor of the model where ε_{CN} is a free parameter. This was done with a likelihood-ratio test.

S2.2.1 Gender

Throughout in the results of the data set we found that there is no significant difference on the 5%-level between women and men concerning condom dispositions. It is therefore of importance to simulate and see if we could detect a difference if there really are one.

We simulated networks with two types of nodes where the nodes only can connect to a node of a different type. The simulated networks are of double size as the data set, $2 \cdot 224$ men and $2 \cdot 422$ women with their degrees. Then we randomly extracted 224 men and 422 women.

On this extracted data we did the analysis first assuming men and women draw their dispositions from the same distribution and then assuming they draw their dispositions from different distributions. With the likelihood-ratio test we compared the likelihoods assuming that men and women draw their dispositions from the same distribution against assuming that they draw them from different distributions. In Table S2.8 we show, for different values of the parameters, how many times we could reject the null hypothesis that men and women draw their dispositions from the same distribution. In S2.9 a summary of the estimates as means and standard deviations are shown. Unless the true distribution of which men and women draw their dispositions from are very similar, we always reject the null hypothesis at the 5% level.

True values							
p_C^M	p_N^M	p_C^W	p_N^W	$\%p < 0.05$	$\%p < 0.01$	$\%p < 0.005$	$\%p < 0.001$
0	0.9	0.7	0.1	100	100	100	100
		0.5	0.2	100	100	100	100
		0.2	0.4	100	100	100	100
0	0.3	0.5	0.2	100	100	100	100
		0.1	0.2	99	93	89	76
0	0.26	0.5	0.2	100	100	100	100
		0.2	0.3	100	100	100	100
		0.05	0.2	59	34	24	11
0.1	0.8	0.5	0.2	100	100	100	100
		0.4	0.4	100	100	100	100
		0.2	0.3	100	100	99	91
0.2	0.3	0.5	0.2	100	100	100	100
		0.4	0.4	100	100	99	99
		0.1	0.2	92	83	78	63

Table S2.8: Simulated configuration network with the degree distribution of the real-world data set. Dispositions and actions were set according to the pro-con-neutral model. It is also assumed that men and women have different disposition distributions. The sample size drawn from the simulated network is $n = 646$ (224 men and 422 females). We did 100 simulations for each parameter set-up and then calculate likelihoods according to the model were men and women have the same disposition distribution and when they have different disposition distributions. We do a likelihood ratio test to see if we can reject the model assuming men and women have the same distribution.

When we study the anal sex dispositions we do at one point only consider the casual sexual relationships, with a sample size of 168 men and 288 females. We did therefore do additional simulations with this sample size, see Table S2.10 and Table S2.11.

True values				Mean and s.d. of estimates							
p_C^M	p_N^M	p_C^W	p_N^W	$\hat{p}_C^M$	sd $\hat{p}_C^M$	$\hat{p}_N^M$	sd $\hat{p}_N^M$	$\hat{p}_C^W$	sd $\hat{p}_C^W$	$\hat{p}_N^W$	sd $\hat{p}_N^W$
0	0.9	0.7	0.1	0.044	0.045	0.325	0.297	0.675	0.059	0.300	0.085
		0.5	0.2	0.014	0.013	0.554	0.315	0.502	0.025	0.397	0.122
		0.2	0.4	0.012	0.026	0.706	0.293	0.226	0.120	0.511	0.240
0	0.3	0.5	0.2	0.009	0.013	0.269	0.089	0.501	0.0245	0.212	0.040
		0.1	0.2	0.004	0.007	0.297	0.050	0.098	0.017	0.204	0.043
0	0.26	0.5	0.2	0.007	0.014	0.244	0.085	0.501	0.021	0.210	0.038
		0.2	0.3	0.004	0.009	0.247	0.061	0.200	0.024	0.311	0.044
		0.05	0.2	0.003	0.006	0.258	0.041	0.050	0.018	0.206	0.038
0.1	0.8	0.5	0.2	0.10	0.036	0.512	0.280	0.500	0.022	0.342	0.177
		0.4	0.4	0.095	0.027	0.510	0.272	0.403	0.025	0.439	0.201
		0.2	0.3	0.097	0.018	0.726	0.163	0.201	0.018	0.322	0.254
0.2	0.3	0.5	0.2	0.199	0.047	0.255	0.147	0.499	0.029	0.206	0.099
		0.4	0.4	0.201	0.036	0.290	0.184	0.398	0.025	0.383	0.109
		0.1	0.2	0.198	0.033	0.294	0.068	0.095	0.021	0.201	0.083

Table S2.9: Estimates of the same simulated samples as in Table S2.8.

True values							
p_C^M	p_N^M	p_C^W	p_N^W	$\%p < 0.05$	$\%p < 0.01$	$\%p < 0.005$	$\%p < 0.001$
0.875	0.125	0.45	0.06	99	98	97	93
		0.3	0.45	100	100	100	100
0.5	0	0.65	0.15	67	49	42	27
		0.3	0.45	89	75	65	55
0.4	0.4	0.65	0.15	98	94	92	86
		0.8	0.1	100	100	100	100
0.3	0.1	0.65	0.15	100	100	100	99
		0.8	0.1	99	99	98	97

Table S2.10: Networks simulated with the same degree distribution as the casual sexual data with dispositions and actions according to the pro-con-neutral model. It is also assumed that men and women have different disposition distributions. The sample size drawn from the simulated network is $n = 456$ (168 men and 288 females). We did 100 simulations for each parameter set-up and then calculate likelihoods according to the model where men and women have the same disposition distribution and when they have different disposition distributions. We do a likelihood ratio test to see if we can reject the model assuming men and women have the same distribution.

True values				Mean and s.d. of estimates							
p_C^M	p_N^M	p_C^W	p_N^W	$\hat{p}_C^M$	sd $\hat{p}_C^M$	$\hat{p}_N^M$	sd $\hat{p}_N^M$	$\hat{p}_C^W$	sd $\hat{p}_C^W$	$\hat{p}_N^W$	sd $\hat{p}_N^W$
0.875	0.125	0.45	0.06	0.865	0.026	0.080	0.061	0.381	0.168	0.168	0.183
		0.3	0.45	0.863	0.027	0.103	0.060	0.268	0.143	0.223	0.255
0.5	0	0.65	0.15	0.472	0.089	0.068	0.104	0.658	0.042	0.110	0.0853
		0.3	0.45	0.495	0.036	0.080	0.120	0.304	0.051	0.391	0.126
0.4	0.4	0.65	0.15	0.373	0.073	0.317	0.175	0.646	0.031	0.139	0.127
		0.8	0.1	0.331	0.136	0.214	0.211	0.793	0.026	0.088	0.078
0.3	0.1	0.65	0.15	0.275	0.078	0.111	0.127	0.641	0.034	0.127	0.081
		0.8	0.1	0.323	0.173	0.185	0.184	0.763	0.070	0.154	0.125

Table S2.11: Estimates of the same simulated samples as in Table S2.10.

S2.2.2 Different relationship types

When simulating data with two different relationship types we need to keep in mind that the dispositions in the two relationships can be the same even if $q = 1$, since the underlying random variable from which we create an ordered sample is a discrete one. An individual draws its disposition for relationship type 1 from X_1 and its disposition for relationship type 2 from X_2. With probability $q(1-(p_C^2+p_I^2+p_N^2))$ the two random variables X_1 and X_2 will not be equal and $X_1 < X_2$. Also,

$$P(X_1 = X_2) = (1-q) + q(p_C^2 + p_I^2 + p_N^2).$$

If $q = 1$ we therefore have that there still is a positive probability, $p_C^2+p_I^2+p_N^2$, that the dispositions can be the same. Therefore, if any of the p_C, p_I or p_N is large there will be a high probability that an individual has the same disposition for both relationship types. It is especially harder to get correct estimates when p_I is very high. This can be seen in Table S2.12 for the set-up $p_C = 0.05$ and $p_N = 0.1$ (and therefore $p_I = 0.85$). We then get the worst, but still a decent, mean estimate for q of 0.96 with a standard deviation of 0.058.

True values		Mean and s.d. of estimates					
p_C	p_N	$\hat{p}_C$	s.d $\hat{p}_C$	$\hat{p}_N$	s.d $\hat{p}_N$	$\hat{q}$	s.d $\hat{q}$
0.05	0.9	0.047	0.008	0.883	0.049	0.983	0.029
	0.5	0.052	0.012	0.505	0.027	0.980	0.028
	0.25	0.053	0.011	0.253	0.017	0.979	0.034
	0.1	0.052	0.012	0.103	0.014	0.961	0.058
0.2	0.7	0.194	0.014	0.684	0.076	0.977	0.032
	0.5	0.200	0.017	0.512	0.052	0.984	0.026
	0.25	0.208	0.016	0.260	0.024	0.976	0.031
	0.1	0.204	0.019	0.107	0.017	0.972	0.044
0.5	0.4	0.492	0.022	0.391	0.079	0.983	0.027
	0.25	0.498	0.022	0.257	0.044	0.987	0.024
	0.1	0.507	0.024	0.112	0.029	0.981	0.034

Table S2.12: Networks simulated with the same degree distribution as the data with dispositions and actions according to the pro-con-neutral model where individuals may have different dispositions depending on which relationship type they have with their sexual partner. The sample size drawn from the simulated network is $n = 646$ (224 men and 422 females). We did 100 simulations for each parameter set-up with the fixed value $q = 1$ and then estimated the parameters.

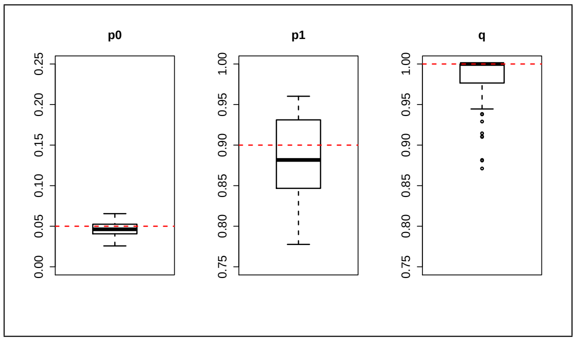

(a) $p_N = 0.9$ and $p_I = 0.05$

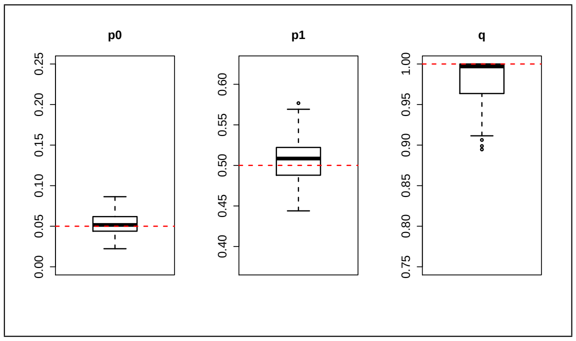

(b) $p_N = 0.5$ and $p_I = 0.45$

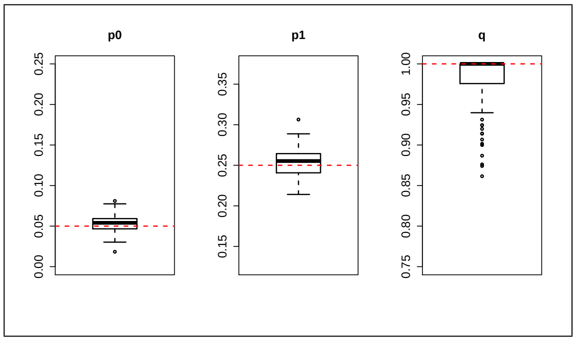

(c) $p_N = 0.25$ and $p_I = 0.7$

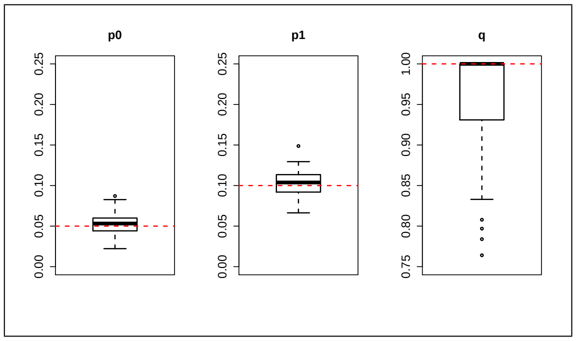

(d) $p_N = 0.1$ and $p_I = 0.85$

Figure S2.1: Boxplots of the estimated from the same simulated data as in Table S2.12 where $p_C = 0.05$, $q = 1$

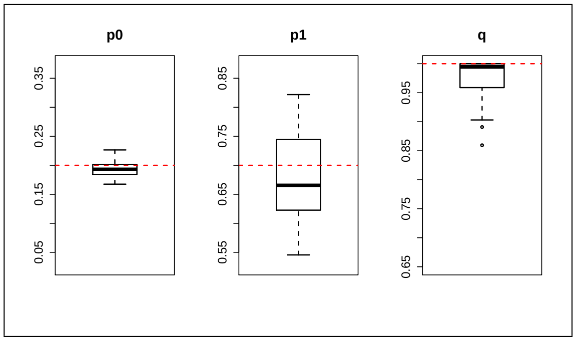

(a) $p_N = 0.7$ and $p_I = 0.1$

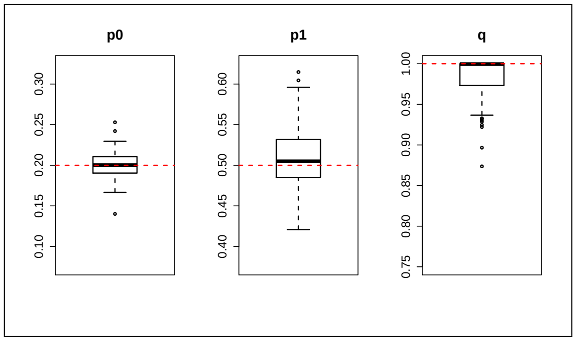

(b) $p_N = 0.5$ and $p_I = 0.3$

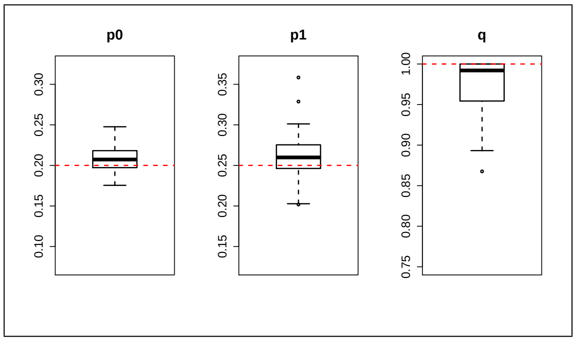

(c) $p_N = 0.25$ and $p_I = 0.55$

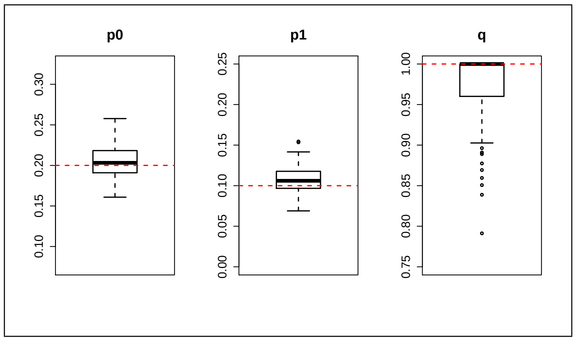

(d) $p_N = 0.1$ and $p_I = 0.7$

Figure S2.2: Boxplots of the estimated from the same simulated data as in Table S2.12 where $p_C = 0.2$, $q = 1$

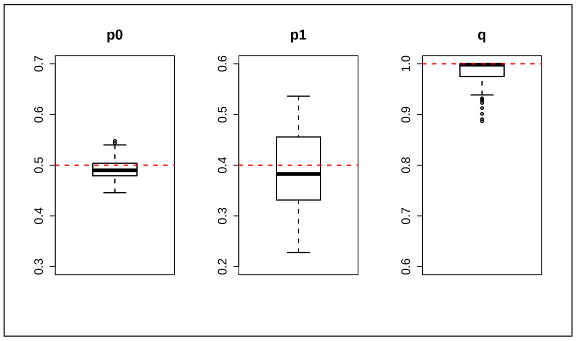

(a) $p_N = 0.4$ and $p_I = 0.1$

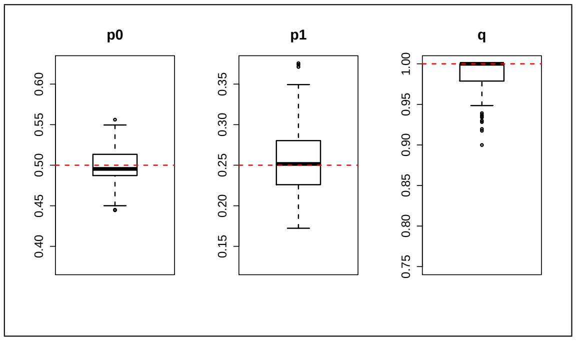

(b) $p_N = 0.25$ and $p_I = 0.25$

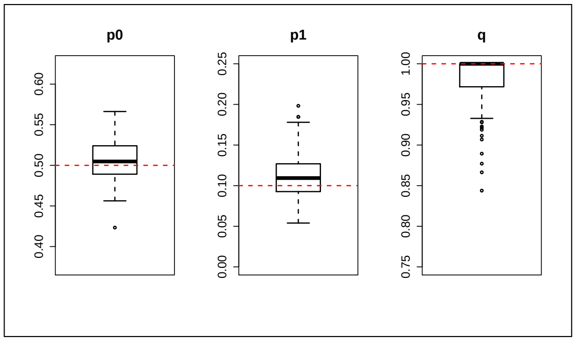

(c) $p_N = 0.1$ and $p_I = 0.4$

Figure S2.3: Boxplots of the estimated from the same simulated data as in Table S2.12 where $p_C = 0.5$, $q = 1$

True values		Mean and s.d. of estimates					
p_C	p_N	$\hat{p}_C$	s.d $\hat{p}_C$	$\hat{p}_N$	s.d $\hat{p}_N$	$\hat{q}$	s.d $\hat{q}$
0.05	0.9	0.048	0.008	0.886	0.059	0.488	0.098
	0.5	0.049	0.011	0.500	0.034	0.495	0.063
	0.25	0.051	0.015	0.250	0.024	0.502	0.072
	0.1	0.051	0.014	0.103	0.018	0.502	0.103
0.2	0.7	0.197	0.017	0.718	0.082	0.505	0.050
	0.5	0.202	0.021	0.510	0.060	0.506	0.050
	0.25	0.201	0.022	0.252	0.032	0.498	0.048
	0.1	0.202	0.022	0.100	0.023	0.503	0.065

Table S2.13: Networks simulated with the same degree distribution as the data with dispositions and actions according to the pro-con-neutral model where individuals may have different dispositions depending on which relationship type they have with their sexual partner. The sample size drawn from the simulated network is $n = 646$ (224 men and 422 females). We did 100 simulations for each parameter set-up with the fixed value $q = 0.5$ and then estimated the parameters.

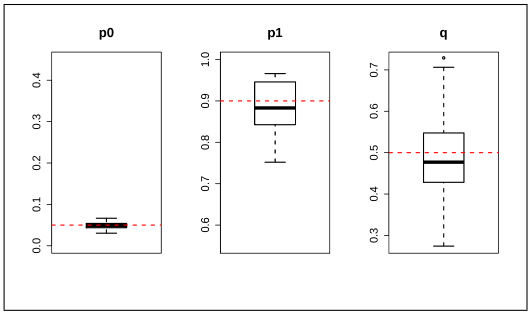

(a) $p_N = 0.9$ and $p_I = 0.05$

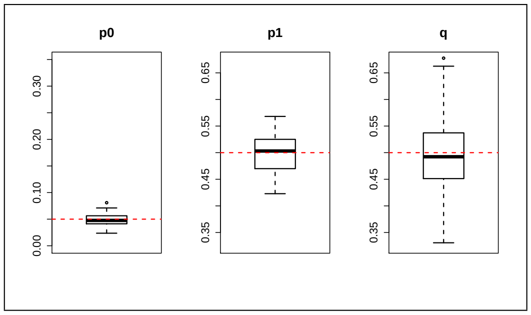

(b) $p_N = 0.5$ and $p_I = 0.45$

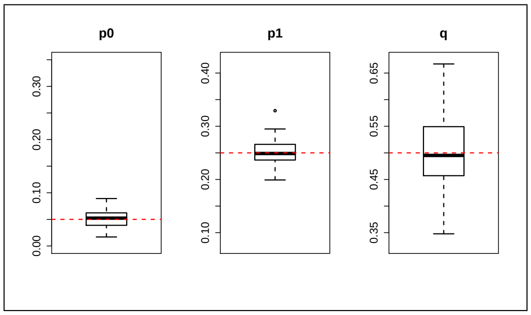

(c) $p_N = 0.25$ and $p_I = 0.7$

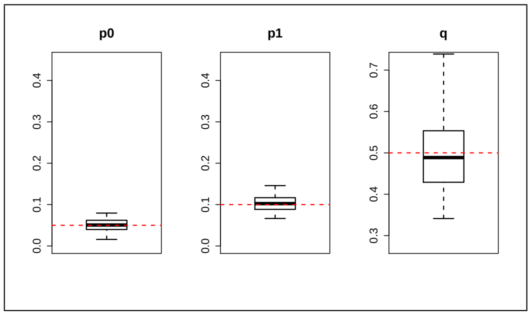

(d) $p_N = 0.1$ and $p_I = 0.85$

Figure S2.4: Boxplots of the estimated from the same simulated data as in Table S2.13 where $p_C = 0.05$, $q = 0.5$

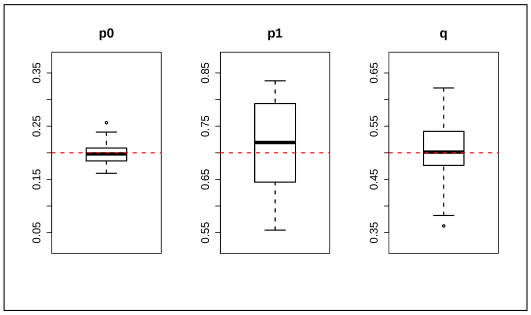

(a) $p_N = 0.7$ and $p_I = 0.1$

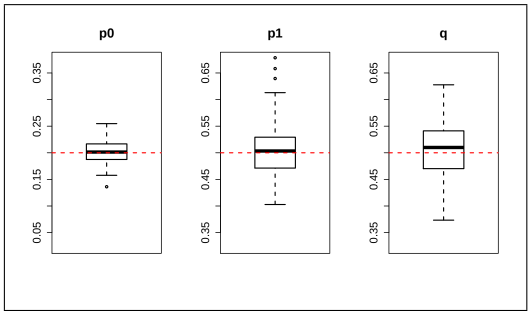

(b) $p_N = 0.5$ and $p_I = 0.3$

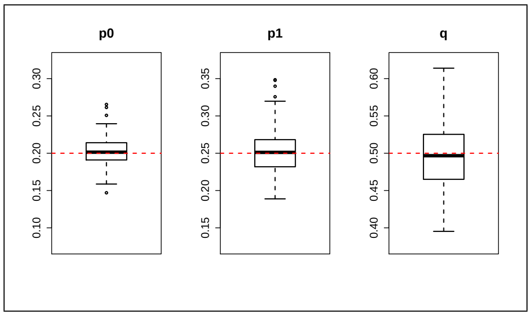

(c) $p_N = 0.25$ and $p_I = 0.55$

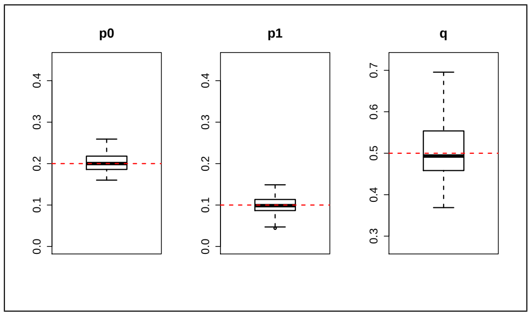

(d) $p_N = 0.1$ and $p_I = 0.7$

Figure S2.5: Boxplots of the estimated from the same simulated data as in Table S2.13 where $p_C = 0.2$, $q = 0.5$

S2.3 Dispositions according to the continuous model

We simulated different networks according to the degree distribution of the real-world data. We then gave each node an individual disposition, x_i, from a known beta distribution. Dependent on the dispositions of two nodes i and j linked together by an edge the edge becomes a condom contact with the probability $1 - \sqrt{x_i x_j}$. From this we again only extracted what should be for us the visible data; how many edges each node has and what type of edge it is, but not to whom and not which dispositions the nodes have. We simulate the network with the same degree distribution as the real-world data.

We begin by simulating a network where men and women have dispositions from the same distribution, but let the parameter γ vary by using the different values $\gamma \in \{0, 0.1, 0.3, 0.4, 0.8\}$. The parameter γ governs who's disposition that weigh more in the decision. If $\gamma = 0.5$ the male disposition weighs as much as the female disposition. If $\gamma = 0$ only the female disposition matters and if $\gamma = 1$ only the male disposition matters.

We test the null hypothesis $\gamma = 0.5$ against that $\gamma \neq 0.5$ for the different networks with true $\gamma \in \{0, 0.1, 0.3, 0.4, 0.8\}$. The percentage of times we reject the null hypothesis for different true parameter values is shown in Table S2.14. We see that unless γ is 0.3 or 0.4 we almost always reject the null hypothesis. In Table S2.15 a summary of the mean and standard deviation of the estimates is shown.

True values						
α	β	γ	$\%p < 0.05$	$\%p < 0.01$	$\%p < 0.005$	$\%p < 0.001$
		0	100	100	100	98
		0.1	100	96	90	86
2	1	0.3	61	44	30	2
		0.4	23	7	4	1
		0.8	89	83	70	50
		0	100	100	100	100
		0.1	100	100	100	100
0.877	0.311	0.3	82	63	56	39
		0.4	30	19	12	4
		0.8	100	98	96	91

Table S2.14: Network simulated with the same degree distribution as the data and assuming men and women have the same disposition distribution but incorporating the parameter γ. The sample size drawn from the simulated network is $n = 646$ (224 men and 422 females). We do 100 simulations for each parameter set-up and calculate likelihoods according to the model where $\gamma = 0.5$ and when γ is a free parameter. We do a likelihood ratio test to see if we can reject the model assuming that $\gamma = 0.5$.

True values			Mean and s.d. of estimates					
α	β	γ	$\hat{\alpha}$	sd $\hat{\alpha}$	$\hat{\beta}$	sd $\hat{\beta}$	$\hat{\gamma}$	sd $\hat{\gamma}$
		0	1.856	0.427	0.921	0.229	0.036	0.057
		0.1	2.004	0.574	0.988	0.320	0.118	0.096
2	1	0.3	2.580	1.173	1.339	0.673	0.249	0.140
		0.4	2.408	1.088	1.240	0.6167	0.351	0.145
		0.8	2.388	0.987	1.237	0.0548	0.829	0.117
		0	0.836	0.137	0.289	0.053	0.016	0.029
		0.1	0.934	0.238	0.338	0.0996	0.088	0.048
0.877	0.311	0.3	0.913	9.230	0.324	0.091	0.300	0.067
		0.4	0.938	0.266	0.339	0.115	0.398	0.071
		0.8	0.934	0.261	0.338	0.109	0.798	0.058

Table S2.15: Estimates of the same simulated samples as in Table S2.14.

Lastly, in this simulation study we test whether we have could detect a difference between gender in disposition distributions. Again we simulate a network with the degree distribution from the data with 422 women and 224 men. We let men and women have dispositions from different distributions and try to estimate the parameters with our models. In Table S2.16 we show the percentage of times we reject the null hypothesis that men and women have dispositions from the same distribution against the alternative hypothesis that they have different disposition distributions. In Table S2.17 a summary of the estimates is shown, the mean and standard deviation of the estimated from the 100 simulations. Here we see that we cannot estimate the parameters correctly.

True values							
α_M	β_M	α_W	β_W	$\%p < 0.05$	$\%p < 0.01$	$\%p < 0.005$	$\%p < 0.001$
2.3	0.8	0.8	2.3	76	52	45	30
		0.2	0.8	100	100	100	100
0.8	0.2	0.2	0.8	100	100	100	100
		4	1	71	49	43	30
1.027	0.276	0.876	0.419	33	17	10	3
		0.1	0.9	100	100	100	100

Table S2.16: Network simulated with the same degree distribution as the data and with different disposition distributions for men and women. The sample size drawn from the simulated network is $n = 646$ (224 men and 422 females). We do 100 simulations for each parameter set-up and calculate likelihoods according to the model where men and women have the same disposition distribution and when they have different disposition distributions. We do a likelihood ratio test to see if we can reject the model assuming that men and women have the same disposition distribution.

True values				Mean and s.d. of estimates							
α_M	β_M	α_W	β_W	$\hat{\alpha}_M$	sd $\hat{\alpha}_M$	$\hat{\beta}_M$	sd $\hat{\beta}_M$	$\hat{\alpha}_W$	sd $\hat{\alpha}_W$	$\hat{\beta}_W$	sd $\hat{\beta}_W$
2.3	0.8	0.8	2.3	15.3	26.5	12.4	22.9	0.776	0.254	2.32	1.52
		0.2	0.8	26.9	34.1	16.8	24.6	0.193	0.039	0.831	0.425
0.8	0.2	0.2	0.8	23.6	34.2	12.1	20.7	0.192	0.035	0.780	0.362
		4	1	0.921	0.811	0.320	0.461	11.9	22.8	3.25	6.04
1.027	0.276	0.876	0.419	2.27	7.99	0.826	3.05	0.879	0.290	0.448	9.27
		0.1	0.9	21.8	32.3	15.0	25.2	0.093	0.023	0.784	0.407

Table S2.17: Estimates of the same simulated samples as in Table S2.16.
