# Supplementary material for: Inferring individual sexual action dispositions from egocentric network data on dyadic sexual outcomes
Source: PLoS One. 2018 Nov 12;13(11):e0207116. doi: 10.1371/journal.pone.0207116 (PMC6231623; doi:10.1371/journal.pone.0207116)
Supplement: S3 Appendix — (PDF) [file pone.0207116.s003.pdf]

Appendix S3: Additional results for first sexual contact and condom dispositions

All results obtained is not presented in the paper. Here we give more thorough results. The results for the three different models (pro-con model, pro-con-neutral model, and continuous model), not assuming different disposition distributions between the genders, are given in Table S3.1.

The pro-con model including the parameter ε_{CN} (the probability of no condom when a condom person meets a non-condom person) is a generalisation of the simpler pro-con model where $\varepsilon_{CN} = 0$. This enables us to do a likelihood ratio test between the two, with the simpler model as a null hypothesis. The null hypothesis that $\varepsilon_{CN} = 0$ is rejected in favour of the model where $\varepsilon_{CN} > 0$ (p-value $< 10^{-6}$). However, when we do the same test between the pro-con-neutral model with both $\varepsilon_{CN} = 0$ and $\varepsilon_{CN} > 0$, we cannot reject the null hypothesis that $\varepsilon_{CN} = 0$ (p-value of 0.08). Also, the pro-con-neutral model with $\varepsilon_{CN} = 0$ fit the data better than the pro-con model with $\varepsilon_{CN} > 0$. Therefore, it seems like we get an improvement of the pro-con model when incorporating ε_{CN} since the model is then more similar to the pro-con-neutral model.

Table S3.1: **Non-condom dispositions: Results assuming females and males draw their disposition from the same distribution**

Model	LogL	AIC	$\hat{p}_N$	$\hat{p}_C$	$\hat{p}_I$	$\hat{\varepsilon}_{CN}$	$\hat{\varepsilon}^{MW}_{CN}$	$\hat{\varepsilon}^{MW}_{NC}$
pro-con	-853.1	1708.2	0.775	0.225	-	-	-	-
pro-con ε_{CN}	-774.9	1553.9	0.628	0.372	-	0.606	-	-
pro-con ε^{MW}	-774.9	1555.9	0.628	0.372	-	-	0.607	0.605
**pro-con-neu**	**-768.0**	**1540.0**	**0.245**	**0.031**	**0.724**	-	-	-
pro-con-neu ε_{CN}	-767.3	1540.6	0.257	0.042	0.701	0.223	-	-
pro-con-neu ε^{MW}	-766.5	1540.9	0.252	0.038	0.710	-	0.465	0
Model	LogL	AIC	$\hat{\alpha}$	$\hat{\beta}$				
continuous	-771.2	1546.5	0.877	0.311				

Maximum Log Likelihood, AIC and parameter estimates for the different models when we assume females and males draw their disposition from the same distribution. p_N stands for the probability of being a non-condom person, p_C of being a condom person and p_I of being neutral. ε_{CN} stands for the probability of non-condom use when a condom person meets a non-condom person. ε^{MW}_{CN} stands for the probability of non-condom use when a male condom person meets a female non-condom person.

The continuous disposition model and the pro-con-neutral model are generalisations of the pro-con model. They are however not generalisations of each other in any way. Therefore, we cannot do a likelihood ratio test to calculate a p-value to reject or keep a model. Instead, AIC scores are used for model comparison. The model with the lowest AIC score fit the data best. In Table S3.1 we list the AIC scores as well as the parameter estimates. The pro-con-neutral model with $\varepsilon_{CN} = 0$ has the lowest AIC score, and it therefore fits the data best. Roughly 25% of all individuals are non-condom individuals, very few are condom individuals (4%) and the rest are neutral according to this model.

The results, assuming men and women have different non-condom disposition distributions, are given in Table S3.2. The continuous disposition model with two different beta distributions, one for men and one women, is not included in the analysis, since with 233 men, simulations showed that we cannot get a good precision for the male beta distribution (not shown).

By either comparing the likelihoods or the AIC score, the pro-con-neutral model fits the data best, which can be seen in Table S3.2 (bold type row). In addition to this, we see that the models that separate by gender, with the exception of the pro-con model, do not provide a better fit. To test whether there is a difference between the genders it is possible to do a likelihood ratio test between each model, not taking gender into account as the null model and the model which does as alternative model. In the pro-con model we can reject the null model that there is no difference between genders ($p-$value 0.008), but this model still does not fit the data better than the pro-con-neutral model assuming no difference between genders. In the other models we cannot reject that there is a difference between genders. If we compare the best fitted

Table S3.2: **Non-condom dispositions: Results assuming females and males draw their disposition from different distributions**

Model	LogL	AIC	$\hat{p}_N^M$	$\hat{p}_N^W$	$\hat{p}_C^M$	$\hat{p}_C^W$	$\hat{p}_I^M$	$\hat{p}_I^W$	$\hat{\varepsilon}_{CN}$	$\hat{\varepsilon}_{CN}^{MW}$	$\hat{\varepsilon}_{NC}^{MW}$
pro-con	-849.6	1703.2	0.754	0.803	0.246	0.198	-	-	-	-	-
pro-con ε_{CN}	-773.5	1553.0	0.384	0.787	0.616	0.213	-	-	0.671	-	-
pro-con ε^{MW}	-772.9	1553.9	0.288	0.810	0.712	0.190	-	-	-	0.698	0.865
**pro-con-neu**	**-766.8**	**1541.6**	**0.256**	**0.204**	**0**	**0.041**	**0.744**	**0.755**	-	-	-
pro-con-neu ε_{CN}	-766.8	1543.6	0.256	0.204	0	0.041	0.744	0.755	0	-	-
pro-con-neu ε^{MW}	-766.4	1544.9	0.252	0.244	0.031	0.039	0.717	0.717	-	0.453	0

Maximum Log Likelihood, AIC and parameter estimates for the different models when we assume females and males draw their disposition from different distributions. p_N stands for the probability of being a non-condom person, p_C of being a condom person and p_I of being neutral. The different superscripts M and F to p_N, p_C and p_I refer to which gender (Male and Female) it belongs to. ε_{CN} stands for the probability of non-condom use when a condom person meets a non-condom person. ε_{CN}^{MW} stands for the probability of non-condom use when a male condom person meets a female non-condom person.

model which assume men and women have the same disposition distribution (pro-con-neutral model in Table S3.1) with the best fitted model which assume men and women have different disposition distributions (also the pro-con-neutral model but in Table S3.2) we get a $p-$value of 0.247.

Next, we estimate the parameters in the continuous disposition model where we have incorporated the parameter γ. This model enables either men or women to be the one whose opinion weighs higher in the decision concerning whether a condom is used or not. The null model is then that $\gamma = 0.5$, which represents an equal weight. If γ is between 0 and 0.5 the woman's disposition weighs higher. We tested the null model against the alternative model where γ is a parameter to be estimated. The null model could not be rejected ($p-$value > 0.3 with likelihood ratio test and γ was estimated to 0.4). Therefore, we cannot reject the model where the dispositions from men and women have equal weight.

Different types of relationships

The data also state which type of relationship each contact was labeled as.

I casual relationship with unknown individual

II casual relationship with known individual

III regular relationship

IV main relationship

There are 429 individuals with main relationships (538 sexual contacts), 264 individuals with regular partners (376 sexual contacts), 368 with a casual known partner (640 sexual contacts) and 252 with an unknown partner (454 sexual contacts). We begin by merging the data into 2 different categories. This merging was done in order to reduce the number of parameters to infer, which is preferable when we have a limited sample size. There are four different types of relationships; there are therefore several different possible mergings. The different combinations are shown in Table S3.3. In all the three mergings we have chosen to keep the order, from casual unknown to main partner, of the relationships. Due to that we want to test if individuals behave differently in more risky contacts than less risky contacts.

Note that the data is of the first sexual contact between two individuals and which relationship type the contact was labeled as. This label could have been decided after the first sexual contact, since the relationship type is usually not known before the first sexual contact between two individuals. The first time two individuals had sex, it is possible that they thought it to be a casual relationship, but it later evolved to be one of the two steady types of relationships. When the relationship type is labeled as one of the casual ones this is not a problem, since then the type of relationship will be the same all the time.

Table S3.3: **Relationship mergings**

Merging name	Category 1	Category 2
1. Casual unknown vs rest	casual unknown	casual known, regular and main
2. Casual vs steady	casual unknown and casual known	regular and main
3. Main vs rest	casual unknown, casual known and regular	main

We merge the four relationship types into two categories. Category 1 always consists of the more risky sexual relationships than category 2. The merging is done to keep the ordering of relationship type, generating three different mergings.

In Table S3.4 we present the result from the analysis with the methods developed to take the different relationship types into account on relationship merging 3, main vs the rest. The analysis was done on all the three different mergings of the data given in Table S3.3, however the results are similar and relationship merging 3 (main partners in one category and the other relationship types in the other category) contains the most significant result. Both the pro-con-neutral model and continuous disposition model which takes into account two different relationships was used. In each of the three mergings of the data, the pro-con-neutral model fit better than the continuous disposition model. More importantly and perhaps surprisingly, individuals seem to use condom more often in relationship category 2 (the category always consisting of more steady partners) than in relationship category 1 (consisting of less steady partners). Further, for the pro-con-neutral models assuming men and women draw their dispositions from different distributions does not give a better fit (Supplementary material S2). Therefore the results for the pro-con-neutral model where gender is not taken into account will be examined a bit closer.

Table S3.4: **Non-condom dispositions: Results when relationship types are taken into account for data merging 3, main vs the rest**

Model	test	Not separated by gender					
		LogL	AIC	$\hat{p}_N$	$\hat{p}_C$	$\hat{p}_I$	$\hat{q}$
pro-con-neutral	$<$	-899.9	1805.8	0.245	0.031	0.724	0
	$>$	-882.9	1771.9	0.232	0.043	0.725	0.363
		LogL	AIC	$\hat{\alpha}$	$\hat{\beta}$	$\hat{q}$	
continuous	$<$	-903.3	1812.6	0.848	0.308	0	
	$>$	-886.6	1779.2	0.749	0.303	0.330	

Results of the analysis on merging 3, main vs the rest, when relationship type is taken into account. $<$ means that the non-condom dispositions is assumed less in category 1 than in category 2, that individuals in category 1 are more likely to use condom compared to individuals in category 2. $>$ means that the non-condom dispositions is assumed greater in category 1 than in category 2, that individuals in category 2 are more likely to use condom compared to individuals in category 1.

For all data mergings and with the assumption that individuals tend to use condom more often in the less steady relationship category, relationship category 1, q is estimated to 0, which means that individuals use the same disposition regardless of relationship category. However, using the model which assumes that individuals tend to use condom *more often* in the more steady relationship category, category 2, q is estimated to be larger than 0 in all three mergings. We cannot compare the AIC scores between the different mergings of the data since these are seen as different data sets. Instead, for all three data mergings, the likelihood ratio test was used to test the null hypothesis that $q = 0$ against the alternative that $q > 0$ for the model where condom is used more often in relationship category 2 (steadier relationship types) than in relationship category 1 (less steady relationship types). For data merging 1, casual unknown vs the rest, we cannot reject the null hypothesis ($p-$value 0.102). For data merging 2, casual vs steady, and 3, main vs the rest, the null hypotheses are rejected with $p-$values of order 10^{-7} and 10^{-9} respectively.

The pro-con-neutral model gives better fit than the continuous geometric mean model (Table S3.4). For

the pro-con-neutral model with the estimates given in Table S3.4, the implication is as follows: probability of condom use in a randomly chosen couple who entered main relationship is 23% higher compared to when we do not take relationship type into account (from 34% to 42%).
A question now arises: since the third merging, where we test the relationship type main against all the other three relationship types, has the lowest $p-$value, is it possible that the second merging where we merge main and regular becomes significant only because those have a main relationship behave differently? We tried to answer this by erasing the sexual contacts which are labeled as main partner and test if individuals tend to use condom more often in the relationship type regular in comparison to the two casual relationship types. We tested this (likelihood ratio test) against the null hypothesis that the dispositions in the two categories are drawn from the same distribution. On the 5 % level we cannot reject the null hypothesis ($p-$value = 0.07). This indicates that the second merging where the two casual relationship types are merged to category 1 and the two steady relationship types are merged into category 2 only becomes significant since those in a main relationship type behaves differently.

Next, we perform an analysis only on the two casual relationships to investigate if there is a difference between known casual and unknown casual. There are 455 individuals with contacts in either or both the casual relationship types. Among these individuals there are 454 sexual contacts in relationship type casual unknown and 640 that are in the casual known relationship type. When only considering the data of the two casual categories we cannot reject the null hypothesis that individuals behave the same in the two casual relationship types, ($p-$value 0.32). We hypothesised that there could have been a difference since individuals could have felt more safe having a casual sexual contact with a known partner than an unknown. Table S3.5 show the estimates and likelihoods.

Table S3.5: **Non-condom dispositions: Results of the analysis done only on the casual relationship types**

	pro-con-neutral					Continuous			
model	LogL	$\hat{p}_N$	$\hat{p}_C$	$\hat{p}_I$	$\hat{q}$	LogL	$\hat{\alpha}$	$\hat{\beta}$	$\hat{q}$
unknown < known	-489.2	0.276	0.005	0.719	0.082	-493.5	1.1228	0.3335	0.082
unknown > known	-489.7	0.280	0.005	0.715	0	-494.0	1.122	0.3300	0

Maximum likelihood and corresponding estimated on the casual relationship data. p_N stands for the probability of being a non-condom person, p_C of being a condom person and p_I of being neutral. α and β are the parameters of the continuous disposition model.

If we only consider the relationship types regular and main we have 560 individuals left with sexual contacts labeled either as regular, main or both. Among these individuals there are 376 sexual contacts in relationship type regular and 538 that are in the main relationship type. When we only study main and regular relationships the estimates can be seen in Table S3.6. The null model is rejected in favour of the model assuming that condom is used more often in main relationships than in regular relationships ($p-$value 0.001). This result follows the same tendency as the ones before; in the first sexual contact individuals tend to use condom *more often* in to-be more steady relationship types.
When we separate the data by both relationship type and gender we do not get a significant better fit (see Supplementary material S2).

Gender: gamma parameter

When we estimate the parameters where we have the same α and β for men and women but incorporate the effect γ, the parameter governing if women's or men's opinion weighs higher, we get the following results. The likelihood not incorporating γ is -770.95, $\alpha = 0.8365$ and $\beta = 0.3041$. The estimate for γ is 0.3948 and the loglikelihood is -769.92 ($\hat{\alpha} = 0.9558$, $\hat{\beta} = 0.3575$). We see that the likelihood is only slightly improved and would for example not be significant with a likelihood ratio test.

Table S3.6: **Non-condom dispositions: Results of the analysis done only on the regular and main relationship types**

	pro-con-neutral					Continuous			
model	LogL	$\hat{p}_N$	$\hat{p}_C$	$\hat{p}_I$	$\hat{q}$	LogL	$\hat{\alpha}$	$\hat{\beta}$	$\hat{q}$
regular $<$ main	-537.5	0.211	0.053	0.736	0	-538.4	0.699	0.314	0
regular $>$ main	-532.2	0.245	0.065	0.690	0.259	-533.6	0.565	0.128	0.239

Maximum likelihood and corresponding estimates on the main and regular relationship data. p_N stands for the probability of being a non-condom person, p_C of being a condom person and p_I of being neutral. α and β are the parameters of the continuous disposition model.

Relationship and gender

In Table S3.3 we presented the different mergings of the relationship data and we give the table below again in Table S3.7. In Table S3.8 the results for the pro-con-neutral model where we use the three different relationship mergings is presented. In Table S3.9 we give the results for the continuous model instead. We did not only separate by relationship types, but gender as well. However, we see that by incorporating gender and assuming that females and males draw their dispositions from different distributions does not have a better fit for any of the three mergings of relationships.

Table S3.7: **Relationship mergings**

Merging name	Category 1	Category 2
1. Casual unknown vs rest	casual unknown	casual known, regular and main
2. Casual vs steady	casual unknown and casual known	regular and main
3. Main vs rest	casual unknown, casual known and regular	main

The three different ways the relationship data is merged into two categories.

Table S3.8: **Non-condom dispositions: Results when relationship types are taken into account using the pro-con-ind model**

		Merged				Separated by Gender						
data	test	LogL	$\hat{p}_C$	$\hat{p}_N$	$\hat{q}$	LogL	$\hat{p}_C^M$	$\hat{p}_C^W$	$\hat{p}_N^M$	$\hat{p}_N^W$	$\hat{q}^M$	$\hat{q}^W$
1	$<$	-876.18	0.031	0.245	0	-874.90	0	0.040	0.252	0.202	0.042	0
	$>$	-874.84	0.031	0.262	0.111	-873.37	0	0.043	0.277	0.218	0.134	0.117
2	$<$	-927.57	0.031	0.245	0	-926.35	0	0.041	0.256	0.204	0	0
	$>$	-914.61	0.036	0.255	0.285	-912.67	0	0.051	0.259	0.222	0.271	0.328
3	$<$	-899.92	0.031	0.245	0	-898.70	0	0.041	0.256	0.204	0	0
	$>$	-882.93	0.043	0.2316	0.363	-880.95	0	0.071	0.251	0.194	0.188	0.523

Results of the analysis on the three different mergings, when relationship type is taken into account. $<$ means that the non-condom dispositions is assumed less in category 1 than in category 2, that individuals in category 1 are more likely to use condom compared to individuals in category 2. $>$ means that the non-condom dispositions is assumed greater in category 1 than in category 2, that individuals in category 2 are more likely to use condom compared to individuals in category 1.

Table S3.9: **Non-condom dispositions: Results when relationship types are taken into account using the continuous model**

		Merged				Separated by Gender						
data	test	LogL	$\hat{\alpha}$	$\hat{\beta}$	$\hat{q}$	LogL	$\hat{\alpha}^M$	$\hat{\beta}^M$	$\hat{\alpha}^W$	$\hat{\beta}^W$	$\hat{q}^M$	$\hat{q}^W$
1	$<$	-879.570	0.852	0.310	0	-878.52	1.095	0.297	0.888	0.425	0	0
	$>$	-877.429	0.809	0.278	0.114	-876.90	1.066	0.275	0.842	0.376	0.119	0.116
2	$<$	-931.018	0.835	0.303	0	-929.904	1.095	0.296	0.892	0.428	0	0
	$>$	-918.39	0.789	0.287	0.269	-917.18	0.993	0.267	0.754	0.356	0.185	0.337
3	$<$	-903.311	0.848	0.308	0	-902.260	1.095	0.296	0.892	0.428	0	0
	$>$	-886.60	0.749	0.303	0.330	-885.383	0.910	0.265	0.782	0.425	0.371	0.345

Results of the analysis on the three different mergings, when relationship type is taken into account. $<$ means that the non-condom dispositions is assumed less in category 1 than in category 2, that individuals in category 1 are more likely to use condom compared to individuals in category 2. $>$ means that the non-condom dispositions is assumed greater in category 1 than in category 2, that individuals in category 2 are more likely to use condom compared to individuals in category 1.
