# Supplementary material for: Inferring individual sexual action dispositions from egocentric network data on dyadic sexual outcomes
Source: PLoS One. 2018 Nov 12;13(11):e0207116. doi: 10.1371/journal.pone.0207116 (PMC6231623; doi:10.1371/journal.pone.0207116)
Supplement: S1 Table — (PDF) [file pone.0207116.s004.pdf]

Table S1: **Anal sex disposition results assuming men and women draw their dispositions from the same distribution**

Model	LogL	AIC	$\hat{p}_A$	$\hat{p}_N$	$\hat{p}_I$	$\hat{\varepsilon}_{NA}$	$\hat{\varepsilon}^{MW}_{NA}$	$\hat{\varepsilon}^{MW}_{AN}$
pro-con	-743.2	1488.5	0.460	0.540	-	-	-	-
pro-con ε_{NA}	-672.5	1348.9	0.137	0.863	-	0.910	-	-
pro-con ε^{MW}	-671.6	1349.1	0.140	0.860	-	-	0.930	0.855
pro-con-neutral	-722.5	1448.9	0.108	0.414	0.478	-	-	-
pro-con-neutral ε_{NA}	-671.8	1349.5	0.115	0.662	0.223	0.903	-	-
pro-con-neutral ε^{MW}	-670.3	1348.5	0.116	0.642	0.242	-	0.940	0.784
Model	LogL	AIC	$\hat{\alpha}$	$\hat{\beta}$	$\hat{\gamma}$			
continuous	-719.1	1442.2	0.111	0.154	-			
continuous γ	-716.3	1438.9	0.262	0.849	0			

Maximum likelihood, AIC and estimate for each model when we assume females and males draw their disposition from the same distribution. A stands for anal sex, N stands for no anal sex and I stands for neutral. For example, p_A is the proportion of individuals who wants to have anal sex. $\hat{\varepsilon}_{NA}$ is the probability for anal sex when a person who wants to have anal sex meets a person who does not. $\hat{\varepsilon}^{MW}_{NA}$ is the probability for anal sex when a male who does not want to have anal sex meets a female who does. $\hat{\varepsilon}^{MW}_{AN}$ is the probability for anal sex when a male who does want to have anal sex meets a female who does.
