# Supplementary material for: Inferring individual sexual action dispositions from egocentric network data on dyadic sexual outcomes
Source: PLoS One. 2018 Nov 12;13(11):e0207116. doi: 10.1371/journal.pone.0207116 (PMC6231623; doi:10.1371/journal.pone.0207116)
Supplement: S2 Table — (PDF) [file pone.0207116.s005.pdf]

Table S2: **Anal sex disposition results assuming females and males draw their disposition from different distributions**

Model	LogL	AIC	$\hat{p}_A^M$	$\hat{p}_A^W$	$\hat{p}_N^M$	$\hat{p}_N^W$	$\hat{p}_I^M$	$\hat{p}_I^W$	$\hat{\varepsilon}_{NA}$	$\hat{\varepsilon}_{NA}^{MW}$	$\hat{\varepsilon}_{AN}^{MW}$
pro-con	-743.2	1490.4	0.464	0.455	0.536	0.545	-	-	-	-	-
pro-con ε_{NA}	-671.4	1348.8	0.125	0.153	0.875	0.847	-	-	0.908	-	-
pro-con ε^{MW}	-671.3	1350.6	0.130	0.150	0.870	0.850	-	-	-	0.919	0.885
pro-con-ind	-717.0	1442.0	0	0.171	0.331	0.468	0.669	0.361	-	-	-
pro-con-ind ε_{NA}	-668.6	1347.2	0.105	0.119	0.296	0.773	0.599	0.108	0.888	-	-
pro-con-ind ε^{MW}	-668.6	1349.2	0.105	0.119	0.297	0.774	0.598	0.107	-	0.889	0.888

Maximum likelihood, AIC and estimates for each model when we assume females and males draw their disposition from different distributions. A stands for pro anal sex, N stands for againt anal sex and I stands for neutral. For example, p_A^M is the proportion men who want to have anal sex. $\hat{\varepsilon}_{NA}$ is the probability for anal sex when a person who wants to have anal sex meets a person who does not. $\hat{\varepsilon}_{NA}^{MW}$ is the probability for anal sex when a male who does not want to have anal sex meets a female who does. $\hat{\varepsilon}_{AN}^{MW}$ is the probability for anal sex when a male who does want to have anal sex meets a female who does.
