# Supplementary material for: Inferring individual sexual action dispositions from egocentric network data on dyadic sexual outcomes
Source: PLoS One. 2018 Nov 12;13(11):e0207116. doi: 10.1371/journal.pone.0207116 (PMC6231623; doi:10.1371/journal.pone.0207116)
Supplement: S3 Table — (PDF) [file pone.0207116.s006.pdf]

Table S3: **Anal sex disposition results for steady relationships assuming men and women draw their dispositions from the same distribution**

Model	LogL	AIC	$\hat{p}_A$	$\hat{p}_N$	$\hat{p}_I$	$\hat{\varepsilon}_{NA}$	$\hat{\varepsilon}_{NA}^{MW}$	$\hat{\varepsilon}_{AN}^{MW}$
pro-con	-480.8	963.6	0.586	0.414	-	-	-	-
pro-con ε_{NA}	-477.7	959.4	0.195	0.805	-	0.95	-	-
pro-con ε^{MW}	-477.7	961.3	0.197	0.803	-	-	0.953	0.926
pro-con-neutral	-486.8	977.6	0.586	0.414	10^{-6}	-	-	-
pro-con-neutral ε_{NA}	-477.7	961.4	0.195	0.804	0.001	0.95	-	-
pro-con-neutral ε^{MW}	-477.6	963.3	0.197	0.802	0.001	-	0.953	0.926

Maximum Log Likelihood for the different models when data is not separated into gender or relationship types. A stands for anal sex, N stands for no anal sex and I stands for neutral. For example, p_A is the proportion of individuals who wants to have anal sex. $\hat{\varepsilon}_{NA}$ is the probability for anal sex when a person who wants to have anal sex meets a person who does not. $\hat{\varepsilon}_{NA}^{MW}$ is the probability for anal sex when a male who does not want to have anal sex meets a female who does. $\hat{\varepsilon}_{AN}^{MW}$ is the probability for anal sex when a male who does want to have anal sex meets a female who does.
