# Supplementary material for: Inferring individual sexual action dispositions from egocentric network data on dyadic sexual outcomes
Source: PLoS One. 2018 Nov 12;13(11):e0207116. doi: 10.1371/journal.pone.0207116 (PMC6231623; doi:10.1371/journal.pone.0207116)
Supplement: S4 Table — (PDF) [file pone.0207116.s007.pdf]

Table S4: **Anal sex disposition results for steady relationships assuming men and women draw their dispositions from different distribution**

Model	LogL	AIC	$\hat{p}_A^M$	$\hat{p}_A^W$	$\hat{p}_N^M$	$\hat{p}_N^W$	$\hat{p}_I^M$	$\hat{p}_I^W$	$\hat{\varepsilon}_{NA}$	$\hat{\varepsilon}_{NA}^{MW}$	$\hat{\varepsilon}_{AN}^{MW}$
pro-con	-486.4	976.8	0.615	0.557	0.385	0.443	-	-	-	-	-
pro-con ε_{NA}	-477.2	960.3	0.174	0.223	0.826	0.777	-	-	0.934	-	-
pro-con ε^{MW}	-476.8	961.6	0.154	0.238	0.846	0.762	-	-	-	0.911	1
pro-con-neutral	-485.7	979.4	0	0.406	0.310	0.406	0.690	0.188	-	-	-
pro-con-neutral ε_{NA}	-477.2	964.3	0.174	0.223	0.826	0.777	0	0	0.934	-	-
pro-con-neutral ε^{MW}	-476.3	964.6	0.146	0.255	0.098	0.745	0.756	0	-	0	1

Maximum Log Likelihood for the different models when data is separated by gender. A stands for anal sex, N stands for no anal sex and I stands for neutral. For example, p_A is the proportion of individuals who wants to have anal sex. $\hat{\varepsilon}_{NA}$ is the probability for anal sex when a person who wants to have anal sex meets a person who does not. $\hat{\varepsilon}_{NA}^{MW}$ is the probability for anal sex when a male who does not want to have anal sex meets a female who does. $\hat{\varepsilon}_{AN}^{MW}$ is the probability for anal sex when a male who does want to have anal sex meets a female who does.
